# A global view of aging and Alzheimer’s pathogenesis-associated cell population dynamics and molecular signatures in human and mouse brains

**DOI:** 10.1038/s41588-023-01572-y

**Published:** 2023-11-30

**Authors:** Andras Sziraki, Ziyu Lu, Jasper Lee, Gabor Banyai, Sonya Anderson, Abdulraouf Abdulraouf, Eli Metzner, Andrew Liao, Jason Banfelder, Alexander Epstein, Chloe Schaefer, Zihan Xu, Zehao Zhang, Li Gan, Peter T. Nelson, Wei Zhou, Junyue Cao

**Affiliations:** 1 Laboratory of Single Cell Genomics and Population Dynamics, The Rockefeller University, New York, NY USA; 2 The David Rockefeller Graduate Program in Bioscience, The Rockefeller University, New York, NY USA; 3 Department of Pathology and Sanders-Brown Center on Aging, University of Kentucky, Lexington, KY USA; 4 The Tri-Institutional MD-PhD Program, New York, NY USA; 5 The Tri-Institutional PhD Program in Computational Biology and Medicine, New York, NY USA; 6 High Performance Computing Resource Center, The Rockefeller University, New York, NY USA; 7 Helen and Robert Appel Alzheimer’s Disease Research Institute, Weill Cornell Medicine, New York, NY USA

**Keywords:** Transcriptomics, Alzheimer's disease

## Abstract

Conventional methods fall short in unraveling the dynamics of rare cell types related to aging and diseases. Here we introduce EasySci, an advanced single-cell combinatorial indexing strategy for exploring age-dependent cellular dynamics in the mammalian brain. Profiling approximately 1.5 million single-cell transcriptomes and 400,000 chromatin accessibility profiles across diverse mouse brains, we identified over 300 cell subtypes, uncovering their molecular characteristics and spatial locations. This comprehensive view elucidates rare cell types expanded or depleted upon aging. We also investigated cell-type-specific responses to genetic alterations linked to Alzheimer’s disease, identifying associated rare cell types. Additionally, by profiling 118,240 human brain single-cell transcriptomes, we discerned cell- and region-specific transcriptomic changes tied to Alzheimer’s pathogenesis. In conclusion, this research offers a valuable resource for probing cell-type-specific dynamics in both normal and pathological aging.

## Abstract (web summary)

Single-cell transcriptomes and single-cell chromatin accessibility profiles generated using EasySci provide a global view of aging and Alzheimer’s pathogenesis-associated cell population dynamics in human and mouse brains.

## Main

Progressive changes in brain cell populations, which can occur during aging, may contribute to functional decline and increased risks for neurodegenerative diseases such as Alzheimer’s disease (AD)^1–4^. Although the recent advances in single-cell genomics have created unprecedented opportunities to explore the cell-type-specific dynamics across the entire mammalian brain^5–8^, most prior studies relied on a relatively shallow sampling of the brain cell populations and failed to reveal rare aging or AD-associated cell types. Additionally, they were technically limited in several ways, including failing to recover isoform-level gene expression patterns and the associated chromatin landscape that regulates cell-type-specific alterations across aging stages.

**Fig. 1:**
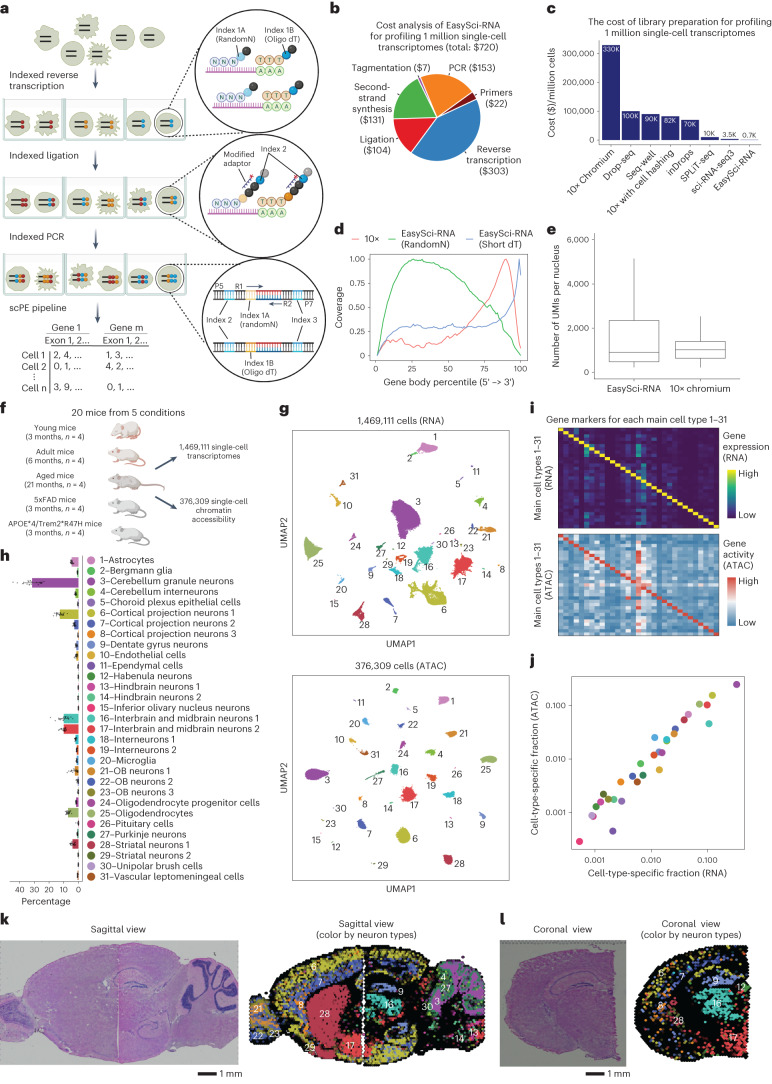
EasySci enables high-throughput and low-cost single-cell transcriptome and chromatin accessibility profiling across the entire mammalian brain. **a**, EasySci-RNA workflow. Key steps are outlined in the texts. scPE, single-cell paired end. **b**, Pie chart showing the estimated cost compositions of library preparation for profiling 1 million single-nucleus transcriptomes using EasySci-RNA. **c**, Bar plot comparing different single-cell RNA-seq methods in terms of their cost of the library preparation for 1 million single-nucleus transcriptomes. The cost of the other techniques (10x Genomics, Drop-seq, Seq-well, inDrops, SPLiT-seq) were calculated using data from previous publications^13,33,83,84^. **d**, Density plot showing the gene body coverage comparing single-cell transcriptome profiling using 10x Genomics and EasySci-RNA. Reads from oligo-dT and random hexamers priming are plotted separately for EasySci-RNA. Short dT, oligonucleotides composed of a stretch of 15 thymine nucleotides. RandomN, oligonucleotides composed of 8 random nucleotides. **e**, Box plot showing the number of unique transcripts detected per mouse brain nucleus comparing 10x Genomics v2 (ref. ^83^) (*n* = 5,351 cells) and an EasySci-RNA library (*n* = 13,440 cells) at similar sequencing depth (~3,800 raw reads per cell). For the box plot, middle lines represent medians, upper and lower box edges represent first and third quartiles, respectively, and whiskers represent 1.5 times the interquartile range (IQR). **f**, Experiment scheme to reconstruct a brain cell atlas of both gene expression and chromatin accessibility across different ages, sexes and genotypes. **g**, UMAP visualization of mouse brain cells by single-cell transcriptome (top) and chromatin accessibility (bottom), colored by main cell types. **h**, Bar plot showing the mean and standard error of the cell-type-specific proportions of the brain cell population across samples (*n* = 20 animals) profiled by EasySci-RNA. **i**, Heatmap showing the aggregated gene expression (top) and gene body accessibility (bottom) of the top 10 marker genes (columns) in each main cell type (rows). **j**, Scatter plot showing the fraction of each cell type in the global brain population by single-cell transcriptome (*x* axis) or chromatin accessibility analysis (*y* axis). **k**,**l**, Mouse brain sagittal (k) and coronal (l) sections showing the H&E staining (left) and the inferred localizations of main neuron types through non-negative least squares (NNLS)-based integration (right), colored by main cell types in **h**.

**Extended Data Fig. 1:**
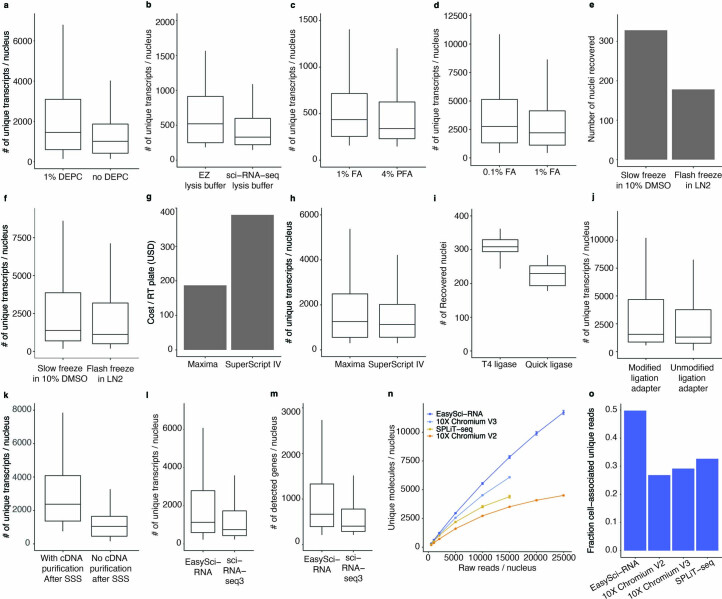
Representative examples showing the performance of optimized conditions of EasySci-RNA. **a-b**. Box plots showing the number of UMIs per nucleus lysed with 1% DEPC (n = 1,596 cells) vs. no DEPC (n = 3,036 cells) in lysis buffer (**a**); or EZ lysis buffer (n = 50 cells) vs. sciRNA-seq3 nuclei lysis buffer (n = 96 cells)^11^ (**b**). **c-d**. Box plot showing the number of UMIs detected per nucleus across fixation conditions: 1% formaldehyde (n = 403 cells) vs 4% paraformaldehyde (n = 358 cells) (**c**); 0.1% formaldehyde (n = 2,952 cells) vs. 1% formaldehyde (n = 2,211 cells) (**d**). **e-f**. Slow freezing condition in 10% DMSO (n = 328 cells) outperformed the flash freezing condition (n = 178 cells) by increasing the number of nuclei recovered (**e**) and the number of UMIs per nucleus (**f**). **g-h**. Maxima reverse transcriptase (n = 5,197 cells) reduces the enzyme cost (**g**) without affecting the number of transcripts detected per nucleus compared to SuperScript™ IV reverse transcriptase (n = 4,071 cells) for profiling mouse brain cells (**h**). **i**. *EasySci-RNA* used T4 ligase (n = 40 wells) instead of quick ligase (n = 16 wells) for a higher recovery rate of nuclei. **j**. Box plot showing the number of unique transcripts detected per nucleus comparing chemically modified ligation primers (n = 499 cells) to unmodified adapters (n = 499 cells). **k**. Additional cDNA purification step after second strand synthesis (n = 90 cells) increased the number of unique transcripts per nucleus compared to without cDNA purification (n = 90 cells). **l-m**. Comparison of the number of unique transcripts and genes in *EasySci-RNA* (n = 11,501 cells) and sciRNA-seq3 (n = 117 cells) with the same sequencing depth ( ~ 2,500 reads/cell) **n**. Line plots showing the median number (with standard error) of unique transcripts per nucleus from a deep sequenced small-scale *EasySci-RNA*, a 10X V2 library^83^, a 10X V3 library and a SPLiT-seq library^33^, all profiling mouse brains (Methods). **o**. Comparison of the fraction of cell-associated unique transcripts from the total number of raw reads across different techniques at the same sequencing depth ( ~ 3,800 raw reads/cell). Boxes in box plots indicate the median and interquartile range (IQR) with whiskers indicating 1.5X IQR.

Here, we introduced EasySci, a cost-effective single-cell profiling strategy based on extensive optimization of single-cell RNA sequencing (RNA-seq) by combinatorial indexing^9^. While the original method has been widely used to study embryonic and fetal tissues^10,11^, it remains restricted to gene quantification proximal to the 3’ end and limited in efficiency and cell recovery rate^11^. EasySci provided improved conditions for cell lysis, fixation, sample preservation, enzymatic reaction, oligonucleotide design, and purification methodologies (Supplementary Table 1). Several test conditions were inspired by optimizations described in recently developed or optimized single-cell techniques^12,13^. The major features of EasySci include (i) 1 million single-cell transcriptomes were prepared for ~US $700 (library preparation cost only, not including personnel or sequencing cost; Fig. 1a–c); (ii) reverse transcription (RT) with indexed oligo-dT and random hexamer primers was achieved, thus recovering cell-type-specific gene expression with full gene body coverage (Fig. 1d); (iii) cell recovery rate, as well as the number of transcripts detected per cell, were substantially improved through optimized nuclei storage, enzymatic reactions and improved primer design (Fig. 1e and Extended Data Fig. 1); and (iv) an extensively improved single-cell data processing pipeline was developed for both gene counting and exonic counting using paired-end single-cell RNA-seq data (Methods).

**Extended Data Fig. 2:**
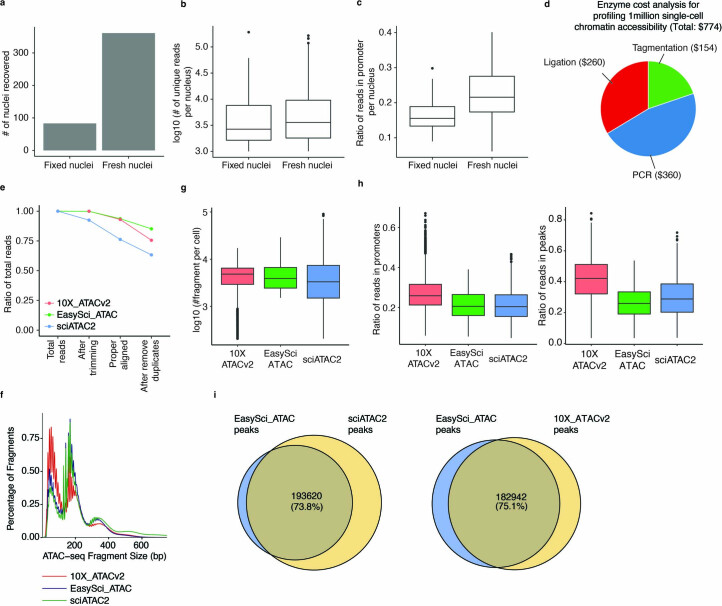
Representative examples showing the performance of optimized conditions of EasySci-ATAC and quality comparison with other single-cell ATAC protocols. **a-c**. We compared two fixation conditions: nuclei were either fixed with 1% formaldehyde for 10 minutes at room temperature (n = 63 nuclei) or directly used for tagmentation without fixation (n = 361 nuclei). The unfixed condition outperformed the fixed condition by increasing cell recovery (**a**), the number of reads (**b**), and the ratio of reads in promoters (**c**) per nucleus. **d**. Pie chart showing the estimated enzyme cost compositions of library preparation for profiling 1 million single-cell chromatin accessibility profiles using *EasySci-ATAC*. **e**. Lineplot showing the ratio of reads loss during each data processing step comparing *EasySci-ATAC*, 10X-ATACv2 and sci-ATAC-seq (referred as ‘sciATAC2’ in the figure). **f**. Histogram showing the fragment length distributions across *EasySci-ATAC*, 10X-ATACv2 and sci-ATAC-seq. **g**. Box plot showing the number of unique fragments comparing *EasySci-ATAC* (n = 3,636 cells), 10X-ATACv2 (n = 6,489 cells) and sci-ATAC-seq (n = 5,494 cells). **h**. Box plots showing the number of reads mapped to promoters (left, defined as ±1 kb around TSS) and peaks (right) comparing *EasySci-ATAC* (n = 3,636 cells), 10X-ATACv2 (n = 6,489 cells) and sci-ATAC-seq (n = 5,494 cells). Peak calling was performed on each dataset separately and peaks were merged to a union peak set. **i**. Pieplot showing the number of peaks that can be repeatedly identified between *EasySci-ATAC* and sci-ATAC-seq (left) and between *EasySci-ATAC* and 10X-ATACv2 (right). Boxes in box plots indicate the median and interquartile range (IQR) with whiskers indicating 1.5X IQR.

Leveraging the technical innovations during the development of EasySci-RNA, we further optimized the single-cell chromatin accessibility profiling method by combinatorial indexing (sci-ATAC-seq3)^14,15^. The key optimizations include (i) a tagmentation reaction with indexed Tn5 that are fully compatible with indexed ligation primers of EasySci-RNA; (ii) a modified nuclei extraction and cryostorage procedure to further increase the library complexity. (A comprehensive quality comparison with other single-cell sequencing assay for transposase-accessible chromatin (scATAC) protocols is shown in Extended Data Fig. 2.) It is noteworthy that the assay for transposase-accessible chromatin with sequencing (ATAC-seq) signal specificity of EasySci-ATAC parallels the original sci-ATAC-seq^14,15^, albeit lower than 10x ATAC-seq, potentially due to the indexed Tn5 used in single-cell combinatorial indexing. The detailed protocols for EasySci are included as supplementary files (Supplementary Protocols 1 and 2) to facilitate individual laboratories to cost-efficiently generate gene expression and chromatin accessibility profiles from millions of single cells.

## Results

### A single-cell catalog of the mouse brain in aging and AD

We first applied EasySci to characterize cell-type-specific gene expression, and chromatin accessibility profiles across the entire mouse brain sampling at different ages, sexes and genotypes (Fig. 1f). We collected C57BL/6 wild-type (WT) mouse brains at 3 months (*n* = 4), 6 months (*n* = 4) and 21 months (*n* = 4). To gain insight into the early molecular changes associated with the pathophysiology of AD, two mutants from the same C57BL/6 background at 3 months were included: an early-onset AD (EOAD) model (5xFAD) that overexpresses mutant human amyloid-beta precursor protein and human presenilin 1 harboring multiple AD-associated mutations^16^; and a late-onset AD (LOAD) model (APOE*4/Trem2*R47H) that carries two of the highest risk factor mutations of LOAD, including a humanized ApoE knock-in allele and missense mutations in the mouse *Trem2* gene^17,18^.

**Extended Data Fig. 3:**
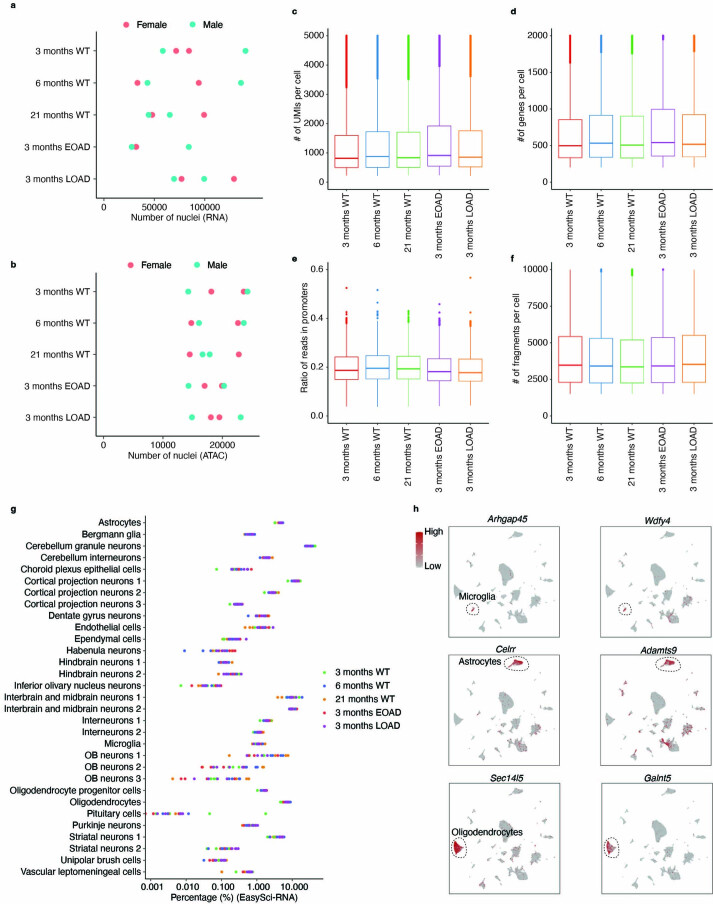
Performance of EasySci-RNA/ATAC profiling of mouse brain samples and identification of main brain cell types and cell-type-specific markers by EasySci-RNA. **a-b**. Scatter plots showing the number of single-cell transcriptomes (**a**) and single-cell chromatin accessibility (**b**) profiled in each mouse individual across five conditions, colored by sex. **c-d**. Box plots showing the number of unique transcripts (**c**) and genes (**d**) detected per nucleus in each condition profiled by *EasySci-RNA* (3-month WT: n = 354,842 cells, 6-month WT: n = 306,537 cells, 21-month WT: n = 257,017 cells, 3-month EOAD: n = 175,673 cells, 3-month LOAD: n = 375,042 cells). **e-f**. Box plots showing the ratio of reads in promoters (**e**) and the number of unique fragments (**f**) per cell in each condition profiled by *EasySci-ATAC* (3-month WT: n = 80,259 cells, 6-month WT: n = 77,101 cells, 21-month WT: n = 71,841 cells, 3-month EOAD: n = 71,548 cells, 3-month LOAD: n = 75,560 cells). **g**. The recovered cell percentage from every main cell type is shown across different replicates. The samples are color-coded based on the condition of origin. **h**. UMAP plots showing the gene expression of identified novel markers for microglia (*Arhgap45*, *Wdfy4*), astrocytes (*Clerr*, *Adamts9*), and oligodendrocytes (*Sec14l5*, *Galnt5*). UMI counts for these genes are scaled by the library size, log-transformed, and then mapped to Z-scores. For all box plots, boxes indicate the median and interquartile range (IQR) with whiskers indicating 1.5X IQR.

In brief, nuclei were extracted from the whole brain and then deposited to different wells for indexed RT (RNA) or transposition (ATAC), such that the first index indicated the originating sample and assay type of any given well. The resulting EasySci libraries (RNA and ATAC) were sequenced separately, yielding a total of 20 billion paired-end reads. After filtering out low-quality cells and doublets, we recovered gene expression profiles in 1,469,111 single nuclei (a median of 70,589 nuclei per brain sample; Extended Data Fig. 3a) and chromatin accessibility profiles in 376,309 single nuclei (a median of 18,112 nuclei per brain sample, Extended Data Fig. 3b) across conditions. Despite shallow sequencing depth (~4,340 and ~16,000 raw reads per cell for RNA and ATAC, respectively), we recovered an average of 1,788 unique molecular identifiers (UMIs) (RNA, median of 935 UMIs) and 5,515 unique fragments (ATAC, median of 3,918) per nucleus (Extended Data Fig. 3c–f), comparable to other published datasets^10,11,14^.

With UMAP visualization^19^ and Louvain clustering^20^, we identified 31 main cell types by gene expression clusters (a median of 16,370 cells per cell type; Fig. 1g), annotated based on cell-type-specific gene markers^2^. Each cell type was present in nearly all individuals, except for rare pituitary cells (0.09% of the population), which were absent in 3 out of 20 individuals (Extended Data Fig. 3g). The cell-type-specific fractions in the global cell population ranged from 0.05% (inferior olivary nucleus neurons) to 32.5% (cerebellum granule neurons) (Fig. 1h). An average of 74 marker genes were identified for each main cell type (defined as at least a twofold expression difference between first- and second-ranked cell types; false discovery rate (FDR) of 5%; and transcripts per million (TPM) > 50 in the target cell type; Supplementary Table 2). In addition to the established marker genes, we identified novel markers that were not previously associated with the respective cell types, such as markers for microglia (e.g., *Arhgap45* and *Wdfy4*), astrocytes (e.g., *Celrr* and *Adamts9*) and oligodendrocytes (e.g., *Sec14l5* and *Galnt5*) (Extended Data Fig. 3h).

**Extended Data Fig. 4:**
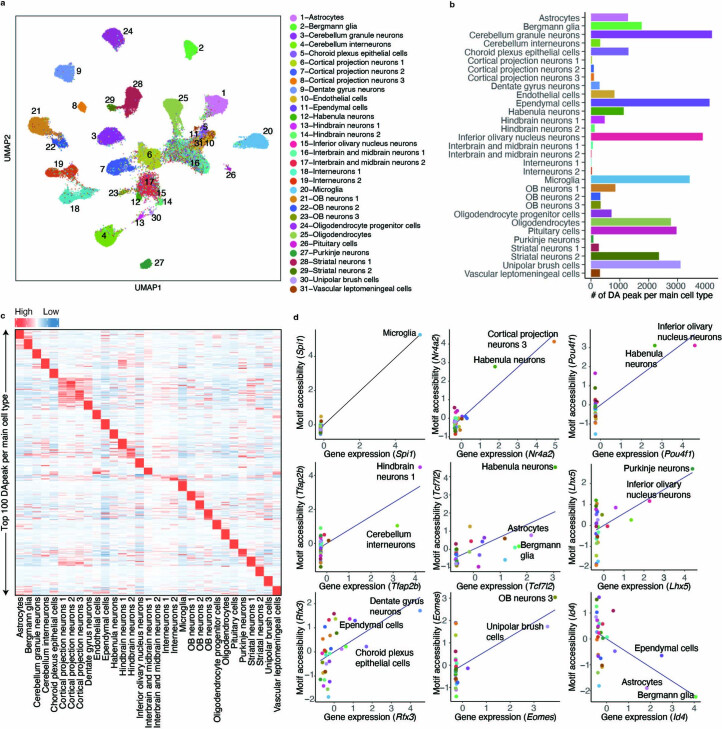
Characterization of cell-type-specific chromatin accessibility and key TF regulators using *EasySci-ATAC*. **a**. UMAP plot of the *EasySci-ATAC* dataset subsampled to 5,000 cells per cell type (or all cells if the number of cells is less than 5,000), colored by main cell types in Fig. 1h. The analysis used the peak-count matrix without integration with the *EasySci-RNA* dataset. **b**. Bar plot showing the number of cell-type-specific peaks for each main cell type (defined as differential accessible (DA) peaks across main cell types with q-value < 0.05 and TPM > 20 in the target cell type). **c**. Heatmap showing the aggregated accessibility of top 100 DA peaks per cell type (ranked by fold change between the maximum and the second accessible cell type). Unique counts for cell-type-specific peaks are first aggregated, normalized by the library size, and then mapped to Z-scores. **d**. Scatter plots showing the correlation between gene expression and motif accessibility of cell-type-specific TF regulators, together with a linear regression line. TF gene expressions are calculated by aggregating scRNA-seq gene counts for each main cluster, normalized by the library size, and then mapped to Z-scores. TF motif accessibilities are quantified by chromVar^101^, then aggregated per main cell type and mapped to Z-scores (Methods).

Several integration analyses were performed to validate the recovered cell types across different layers. First, we applied a deep-learning-based strategy^21^ to integrate transcriptome and chromatin accessibility profiles, yielding 31 main cell types (Fig. 1g). The gene body accessibility and expression of marker genes across cell types were highly correlated (Fig. 1i), as well as the fraction of each cell type (Pearson correlation *r* = 0.95, *P* = 6.68 × 10^−16^) (Fig. 1j). We further investigated the epigenetic controls of the diverse brain cell types through differential accessibility analysis (Extended Data Fig. 4a). We identified a median of 474 differential accessible peaks per cell type (FDR of 5%, TPM > 20 in the target cell type; Extended Data Fig. 4b,c and Supplementary Table 3). Key cell-type-specific transcription factor (TF) regulators were discovered by correlation analysis between motif accessibility and expression patterns, such as *Spi1* in microglia^22^, *Nr4a2* in cortical projection neurons 3 (ref. ^23^) and *Pou4f1* in inferior olivary nucleus neurons^24^ (Extended Data Fig. 4d).

We next integrated our dataset with a 10x Visium spatial transcriptomics dataset through a modified NNLS approach (Methods). As expected, specific brain cell types were mapped to distinct anatomical locations (Fig. 1k,l), especially for region-specific cell types such as cortical projection neurons (clusters 6–8), cerebellum granule neurons (cluster 3) and hippocampal dentate gyrus neurons (cluster 9). These integration analyses confirmed the annotations and spatial locations of main cell types in our single-cell datasets.

### In-depth view of cellular subtypes in the mammalian brain

**Fig. 2:**
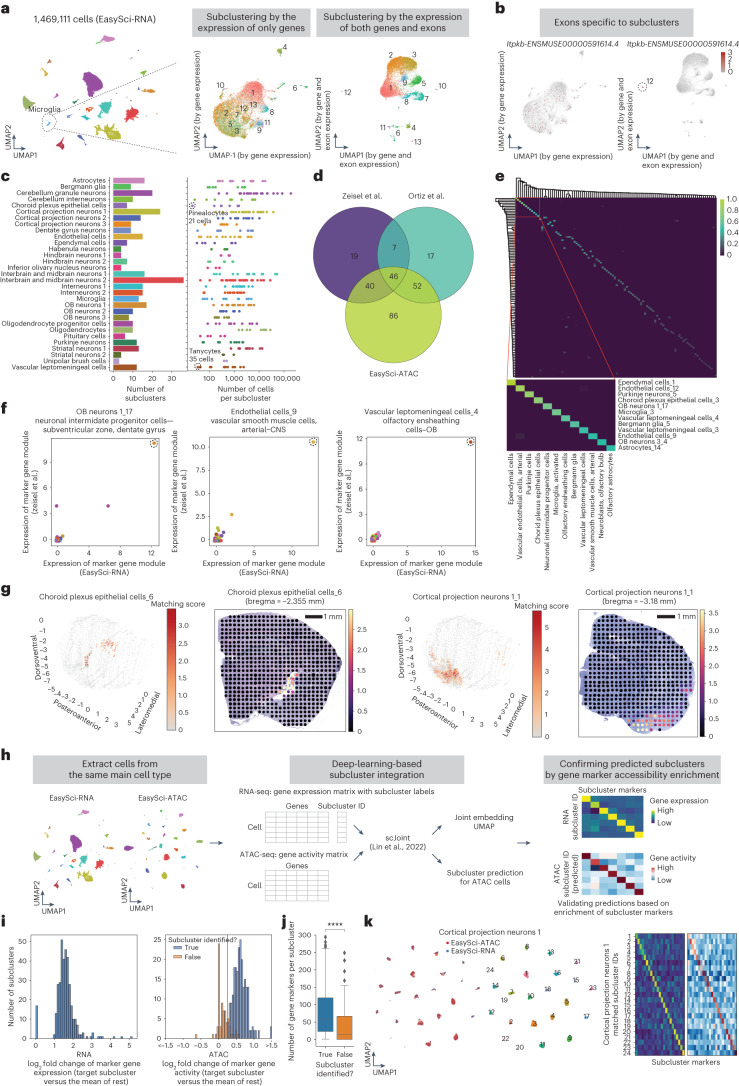
Identification of cellular subtypes in the mouse brain. **a**, Example UMAPs of microglia cells subjected to subclustering analysis based on gene expression alone (middle) or both gene and exon expression (right), colored by subcluster ID derived from combined gene and exon information. **b**, UMAP plots same as **a**, showing the expression of an exonic marker *Itpkb-ENSMUSE00000591614.4* of microglia subcluster 12. **c**, For each main cell type, bar plot showing the number of subclusters (left) and dot plot showing the number of cells from each subcluster (right). Two rare subclusters (choroid plexus epithelial cells-7 and vascular leptomeningeal cells-2) are circled. **d**, Venn diagram showing the number of validated subclusters using integration analysis with Zeisel et al.^2^, Ortiz et al.^28^ or the EasySci-ATAC dataset. **e**, Heatmap illustrating the similarity score between cell types derived from EasySci (rows) and Zeisel et al.^2^ (columns), colored by the min-max normalized beta values obtained from cell-type correlation analysis (Methods). **f**, Dot plot showing the expression of example GMs unique to subclusters across paired cell types between the EasySci dataset and Zeisel et al.^2^, colored by main cell types (same as Fig. 2c). **g**, Spatial distributions of example subclusters, inferred using a brain spatial transcriptomics atlas^28^ and the cell2location approach^27^, colored by matching score. **h**, Scheme outlining scRNA and scATAC integration analyses. **i**, Histogram showing the log_2_ fold change of marker gene expression from RNA-seq (left) and marker gene activity from ATAC-seq (right) per subcluster compared to the rest of cells. Predicted subclusters with log_2_ fold change of gene activity > 0.25 between the target subcluster and the rest of cells and contain more than 10 cells are considered matched. **j**, Box plots illustrate the number of markers comparing the subclusters that could be validated in ATAC-seq data (*n* = 224 subclusters) to those that could not (*n* = 135 subclusters). Two-sided Wilcoxon rank-sum test was used. ****, *P* < 0.0001 (*P* = 7.8 × 10^−8^). **k**, Example subcluster integration results of cortical projection neurons 1. Left: UMAP plots demonstrating the overlap of two molecular layers, color-coded by subcluster ID Right: heatmaps showing the enrichment of gene activity and gene accessibility of matching subclusters.

**Extended Data Fig. 5:**
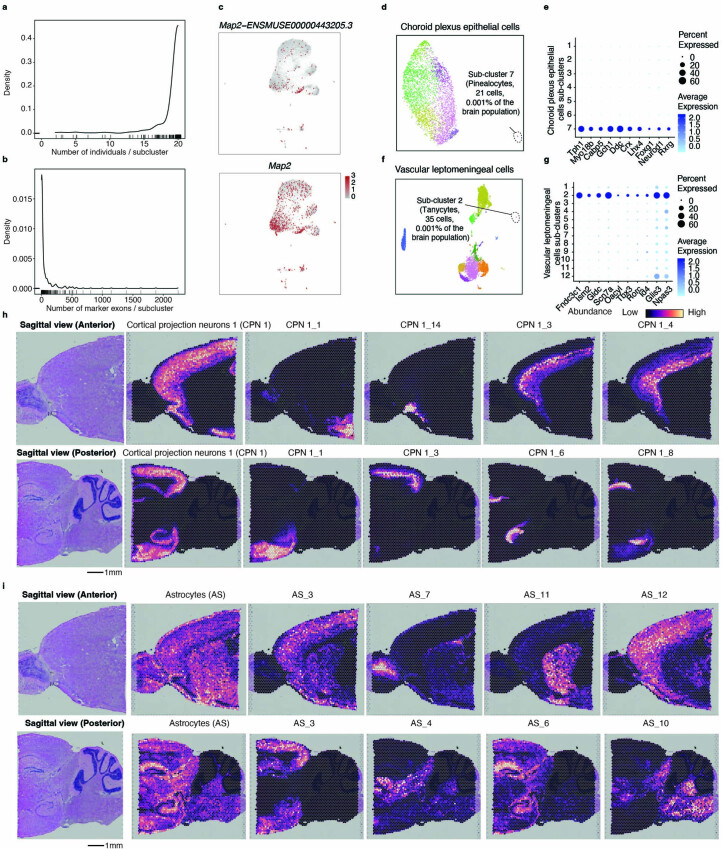
Characterizations of cellular subtypes in the mouse brain. **a**. Density plot showing the number of individuals per subcluster. The rug plot below the density plot represents the individual subclusters. **b**. Density plot of the number of marker exons per subcluster. The rug plot below the density plot represents the individual subclusters. **c**. UMAP plots, same as Fig. 2a based on both gene and exon-level expression, showing the specific expression of an example exon marker *Map2-ENSMUSE00000443205.3* (top) of microglia subcluster 8 and the lack of specificity of its corresponding gene *Map2* (bottom). Single-cell gene/exon expression was normalized first by library size, log-transformed, and then scaled to Z-scores. **d**. UMAP visualizations showing subclustering analysis for choroid plexus epithelial cells colored by subcluster IDs, highlighting the rare subcluster shown in Fig. 2c. **e**. Dot plot showing the expression of selected marker genes for choroid plexus epithelial cells-7, including both normal genes (left five genes) and transcription factors (right five genes). **f**. UMAP visualizations showing subclustering analysis for vascular leptomeningeal cells colored by subcluster IDs, highlighting the rare subclusters shown in Fig. 2c. **g**. Dot plot showing the expression of selected marker genes for vascular leptomeningeal cells-2, including both normal genes (left five genes) and transcription factors (right five genes). **h-i**. Mouse brain sagittal sections showing spatial abundances of main cell types and related subclusters for cortical projection neurons 1 (**h**) and astrocytes (**i**) in anterior (top) and posterior (bottom) regions, estimated using the cell2location^27^.

Rather than performing subclustering analysis with the gene expression alone, we exploited the unique feature of EasySci-RNA (that is, full gene body coverage) by incorporating both gene counts and exonic counts for principal-component analysis followed by unsupervised clustering. The approach substantially increased the clustering resolution, as shown in a microglia subtype example (Fig. 2a,b). Leveraging this subclustering strategy, we identified a total of 359 subclusters, with a median of 1,038 cells in each group (Fig. 2c). All subclusters were contributed by multiple individuals, with a median of nine exonic markers enriched in each subcluster (Extended Data Fig. 5a,b and Supplementary Table 4). Some subtype-specific exonic markers were not detected by conventional differential gene analysis (for example, *Map2-ENSMUSE00000443205.3* in microglia-8; Extended Data Fig. 5c). Notably, our strategy favors detecting extremely rare cell types, such as rare pinealocytes (choroid plexus epithelial cells 7, 21 cells, marked by *Tph1* and *Ddc*^25^) and tanycytes (vascular leptomeningeal cells-2, 35 cells, marked by *Fndc3c1*, *Scn7a*^26^) (Extended Data Fig. 5d–g).

**Extended Data Fig. 6:**
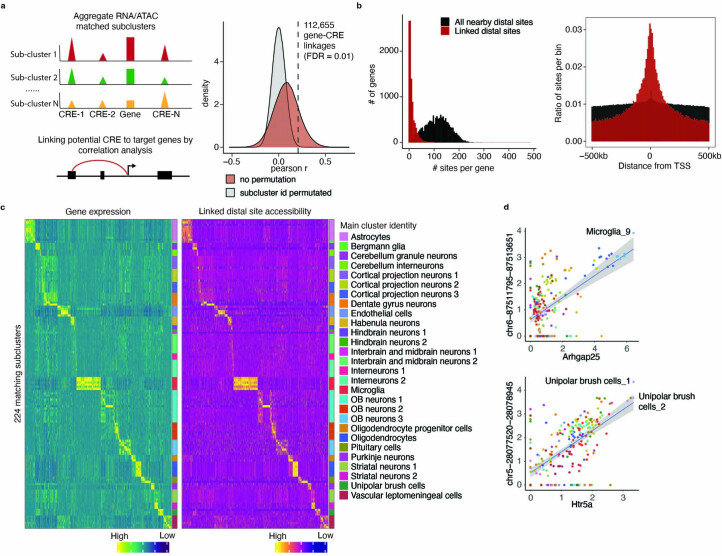
Cis-regulatory elements regulation of gene expression at the subcluster level. **a**. Overview of analytical steps (left) and density plot showing the distribution of Pearson correlation coefficients between gene expression and the accessibility of distal linked site (colored in red) or all nearby accessible elements (within ±500 kb of the promoter, colored in gray) across pseudo-cells. Dash line indicates the Pearson correlation coefficient cutoff with 0.01 FDR. **b**. Left, histogram showing the number of accessible sites per gene. Right, histogram shows the distance distribution of accessible sites within 500 kb of genes. Both plots include all nearby accessible sites (colored in black) and the linked accessible sites (colored in red). **c**. Heatmaps showing the concordant expression of genes (left) and accessibility of putative distal linked sites (right) across all matched subclusters. Rows represent the aggregated gene expression or peak accessibility for a given subcluster. The raw aggregated RNA-seq and ATAC-seq data was normalized first by the total number of reads for each subcluster and then scaled to Z-score across all subtypes. **d**. Correlation plot showing examples of subcluster-specific genes and linked accessible sites for microglia-9 and unipolar brush cells-2. Gray area indicates 95% confidence intervals around the linear regression line.

About 75% of the 359 cell subclusters can be validated through integration analysis with other datasets (Fig. 2d). Our initial integration with a single-cell dataset featuring highly detailed cell type annotations^2^ enables the validation of 112 subclusters, each matching with cell types documented in the previous study^2^ (Fig. 2e and Supplementary Table 5). These corresponding cell types were further validated by their cell-type-specific markers, exemplified by neuronal intermediate progenitor cells, vascular smooth muscle cells, and olfactory ensheathing cells (Fig. 2f). Next, we integrated the 10x Visium spatial transcriptomics datasets^27^ and determined the region-specificity of the recovered cell types or subtypes^27^ (Extended Data Fig. 5h,i). We then expanded the analysis to include an extensive spatial transcriptomics dataset encompassing 75 coronal sections of the mouse brain^27,28^ and discovered 122 subclusters with high spatial mapping scores (Supplementary Table 5 and Methods). For instance, our analysis revealed that choroid plexus epithelial cells-6 were primarily situated in the lateral ventricle, whereas cortical projection neurons 1-1 were predominantly found in the amygdala (Fig. 2g). As the third approach to confirm these subclusters, we utilized a deep-learning-based method^21^ to integrate the snRNA-seq and snATAC-seq data from each main cell type and recovered 224 ‘corresponding subclusters’ between the two molecular layers (Fig. 2h,i). As expected, the subclusters validated by ATAC-seq data exhibit more markers than those not validated (Fig. 2j). For example, the chromatin landscape for all 24 subclusters from cortical projection neurons 1 cells was recognized and validated by the significant enrichment of marker gene expression and activity in the target subcluster (Fig. 2k). We further explored *cis*-regulatory elements at the cell subtype resolution by correlation-based linkage analysis and unveiled a global network of putative enhancer-gene pairs shaping brain cell heterogeneity (Extended Data Fig. 6).

**Extended Data Fig. 7:**
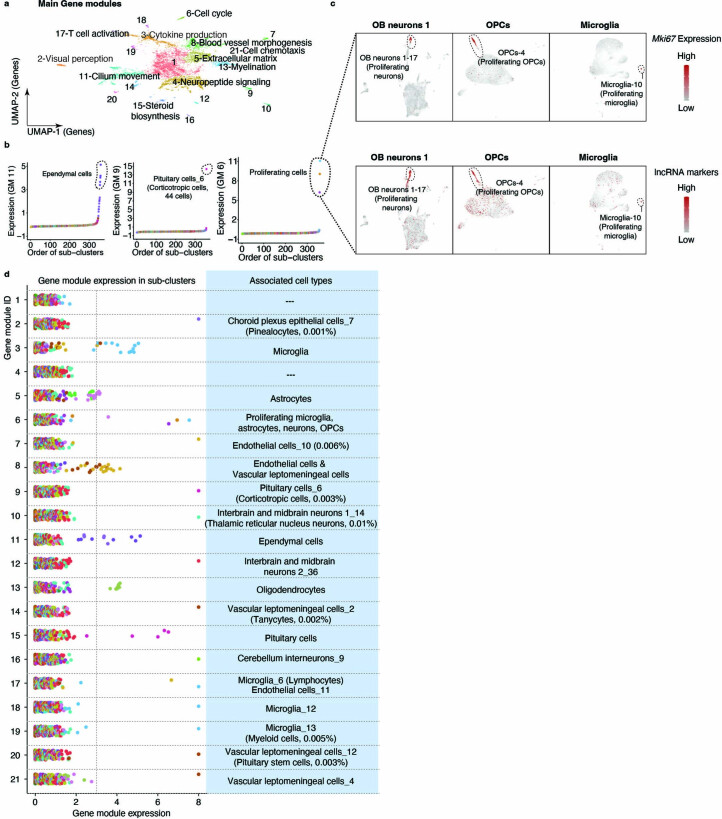
Identification of key molecular programs underlying cell type specificity in the mouse brain. **a**. UMAP visualizations of genes colored by identified gene module IDs. **b**. Scatter plots showing examples of gene modules and their expression levels across subclusters (ordered by the level of gene module expression): GM-11 is specific to ependymal cells; GM-9 is specific to pituitary cell-6 (corticotropic cells); GM-6 marks four proliferating subclusters from different main cell types. UMI counts for genes from each gene module are scaled for library size, log-transformed, mapped to Z-scores and then aggregated. **c**. UMAP visualization showing three proliferating subclusters identified from OB neurons 1, oligodendrocyte progenitor cells, and microglia, colored by the normalized expression of canonical proliferating marker *Mki67* (top) and the aggregated expression of lncRNAs in GM-6 (bottom). UMI counts are first normalized by library size, log-transformed, and then mapped to Z-scores. OPCs, oligodendrocyte progenitor cells. **d**. Scatter plot showing the expression of each gene module across 359 subclusters. The associated cell types were annotated on the plot. UMI counts for genes from each gene module are scaled for library size, log-transformed, mapped to Z-scores and then aggregated.

We next investigated key molecular programs underlying diverse cellular subtypes by clustering genes based on their expression variance across all 359 cell subclusters (Extended Data Fig. 7). We identified 21 gene modules (GMs), with the largest one (GM1) corresponding to a group of housekeeping genes. Several GMs were enriched in specific cell subtypes, such as the ependymal cell-specific GM^29^ (GM11), and pituitary cells subtype-6 specific GM (GM9)^30^. Similar analysis revealed programs in other rare subtypes, such as microglia-13 (GM19), vascular leptomeningeal cells-12 (GM20) and choroid plexus epithelial cells-7 (GM2). Remarkably, rare proliferating cells were identified through a cell-cycle-related GM (GM6), which include both conventional proliferating markers (for example, *Mki67)*, and a group of less-studied lncRNAs (for example, *Gm29260* and *Gm37065*) (Extended Data Fig. 7c and Supplementary Table 6).

### Aging-associated population dynamics at subtype resolution

**Fig. 3:**
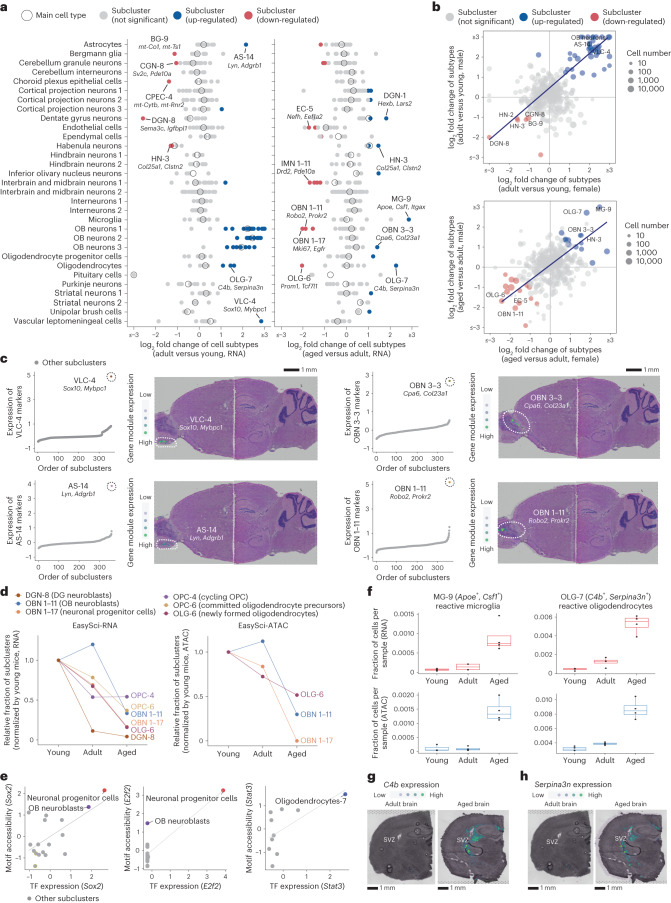
Identifying brain cell population changes across the lifespan at subtype resolution. **a**, Dot plots showing the log-transformed cell-type-specific fraction changes of main cell types (circles) and subclusters (dots) between adult versus young (left) and between aged versus adult (right) from EasySci-RNA data. Significantly changed subclusters were colored by the direction of changes. Representative subclusters were labeled along with top gene markers. AS, astrocytes; BG, Bergmann glia; CGN, cerebellum granule neurons; CPEC, choroid plexus epithelial cells; DGN, dentate gyrus neurons; EC, endothelial cells; HN; habenula neurons; IMN 1, interbrain and midbrain neurons 1; MG, microglia; OBN 1, OB neurons 1; OBN 3; OB neurons 3; OLG, oligodendrocytes; OPC, oligodendrocyte progenitor cell; VLC; vascular leptomeningeal cells. **b**, Scatter plots showing the correlation of the subcluster specific fraction changes between males and females in the early growth stage (top) and the aging stage (bottom), with a linear regression line. The most significantly changed subclusters are annotated on the plots. **c**, Examples of development- or aging-associated subclusters and their spatial positions. Left: scatter plots showing the aggregated expression of subcluster-specific marker genes across all subclusters. Right: plots showing the aggregated expression of subcluster-specific marker genes across a brain sagittal section in 10x Visium spatial transcriptomics data. **d**, Line plots showing the relative fractions of depleted subclusters across three age groups identified from EasySci-RNA (left) and EasySci-ATAC (right). **e**, Scatter plots showing the correlated gene expression and motif accessibility of TFs across subclusters enriched in OB neurons 1–17 (*Sox2* and *E2f2*, left and middle) and oligodendrocytes-7 (*Stat3*, right), together with a linear regression line. **f**. Box plots showing the fractions of the reactive microglia (left) and reactive oligodendrocytes (right) across three age groups (young: n = 4 mice, adult: *n* = 4 mice, aged: *n* = 4 mice) profiled by EasySci-RNA (top) and EasySci-ATAC (bottom). **g**,**h**, Mouse brain coronal sections showing the expression level of *C4b* (g) and *Serpina3n* (h) in the adult (left) and aged (right) brains from spatial transcriptomics analysis. Boxes in box plots indicate the median and IQR with whiskers indicating 1.5× IQR.

To obtain a global view of brain cell population dynamics across the adult lifespan, we first quantified the cell-type-specific fractions recovered from each individual mouse. Differential abundance analyses were conducted across all 359 subclusters, yielding 45 and 29 significantly changed subclusters during early growth (between 3 and 6 months) and aging (between 6 and 21 months; Fig. 3a and Supplementary Tables 7 and 8), respectively. Significantly changed cell subtypes were strongly correlated between genders (Fig. 3b).

Consistent with the growth of the olfactory bulb (OB) during early development^31^, we observed significant expansion in all OB neuron subtypes during this phase. Meanwhile, a rare astrocyte subtype (AS-14, *Lyn*^*+*^ *Adgrb1*^+^) and a vascular leptomeningeal cell subtype (VLC-4, *Sox10*^*+*^ *Mybpc1*^+^) exhibited significant expansion in the same period (Fig. 3c). AS-14 featured with genes (for example, *BAI1*) involved in the clean-up of apoptotic neuronal debris produced during brain fast growth^32^, and VLC-4 highly expressed genes (for example, *Sox10* and *Mybpc1* (refs. ^33,34^)) involved in the growth of axons^35^. Both subclusters were spatially mapped to the OB region, suggesting their potential involvement in OB expansion (Fig. 3c). In contrast to the early growth stage, most OB neurons remained relatively stable during aging, with only a few subtypes showing significant changes. Key examples include the expansion of an OB neuron subtype corresponding to excitatory neurons in the mitral cell layer of the OB region^36^ (OBN 3–3, marked by *Cpa6* and *Col23a1*), and the depletion of OB neuroblasts^2,37^ (OBN 1–11, marked by *Robo2* and *Prokr2*). Integration analysis with spatial transcriptomics datasets indicate these cell types were mapped to different regions of the OB (Fig. 3c).

**Extended Data Fig. 8:**
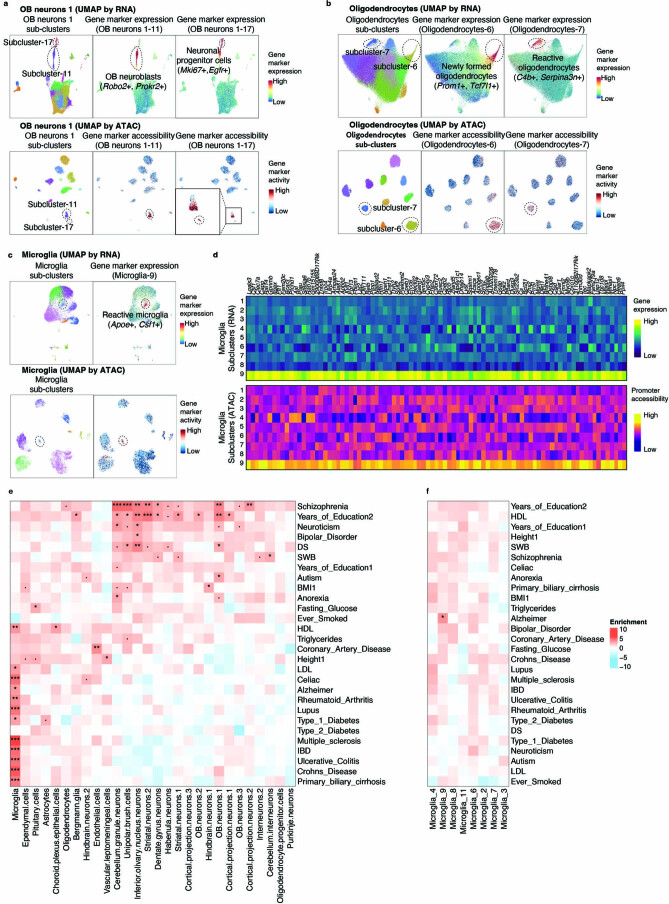
Identifying aging-associated subclusters in *EasySci-ATAC* and cell-type-specific enrichment of GWAS heritability of human phenotypes. **a**. UMAP visualization showing OB neurons 1-11 and OB neurons 1-17 identified from *EasySci-RNA* (top) and *EasySci-ATAC* (bottom), colored by subcluster id (left), aggregated gene expression or gene activity of OB neurons 1-11 gene markers (middle) and OB neurons 1-17 gene markers (right). **b**. UMAP visualization showing oligodendrocytes-6 and oligodendrocytes-7 identified from *EasySci-RNA* (top) and *EasySci-ATAC* (bottom), colored by subcluster id (left), aggregated gene expression or gene activity of oligodendrocytes-6 gene markers (middle) and oligodendrocytes-7 markers (right). **c**. UMAP visualization showing microglia-9 identified from *EasySci-RNA* (top) and *EasySci-ATAC* (bottom), colored by subcluster id (left), aggregated gene expression or gene activity of microglia-9 gene markers (right). Subcluster marker genes were identified by differential expression analysis using scRNA-seq data (Methods). **d**. Heatmap showing the gene expression (top) and the promoter accessibility (bottom) of microglia-9 enriched genes across subclusters. The *EasySci-RNA* data (UMI count matrix) and *EasySci-ATAC* data (read count matrix) were aggregated per subcluster, normalized by the total number of reads, column centered, and scaled. Of note, rare subclusters from RNA-seq data that were not detected in ATAC-seq data were not included in this analysis. **e-f**. Heatmap showing the results of LDSC analysis of the SNPs associated with the indicated phenotypes in DEpeaks across main cell types (**e**) or across microglia subtypes (**f**), colored by z score of regression coefficient. *FDR < 0.05, **FDR < 0.01, ***FDR < 0.001. Only cell types that contain DEpeaks from all autosomes are included in this analysis.

More than twenty brain cell subtypes showed a marked reduction across the adult lifespan. For example, the most depleted populations in the aged brain include OB neuroblasts (OBN 1–11, marked by *Prokr2* and *Robo2*^2,37^), OB neuronal progenitor cells (OBN 1–17, marked by *Mki67* and *Egfr*^38^), and dentate gyrus neuroblasts (DGN-8, marked by *Sema3c* and *Igfbpl1*^39^) (Fig. 3d). DG neuroblasts declined even in the early growth, suggesting an earlier decline of DG neurogenesis compared to OB neurogenesis. In contrast to age-associated depletion of neurogenesis progenitors, oligodendrocyte progenitors (OPC-4, marked by *Pdgfra* and *Mki67*) remained relatively stable. However, newly formed oligodendrocytes (OLG-6, marked by *Prom1* and *Tcf7l1* (ref. ^38,40^)) and committed oligodendrocyte precursors (OPC-6, marked by *Bmp4* and *Enpp6* (ref. ^38,40,41^)) decreased during aging, indicating impaired oligodendrocyte differentiation. The age-associated population dynamics were further validated using the scATAC-seq dataset (Fig. 3d and Extended Data Fig. 8a,b) and our companion study in which we tracked cell dynamics via metabolic labeling^42^. Furthermore, we identified subtype-specific TF regulators using both gene expression and TF motif accessibility. This includes recognized regulators of neurogenesis (for instance, *Sox2* and *E2f2*^43,44^), demonstrating the potential of our datasets to unveil key epigenetic signatures of aging-associated cell subtypes (Fig. 3e).

A total of 14 cell subtypes notably expanded in the aged brain, such as a microglia subtype (MG-9, *Apoe*^+^, *Csf1*^+^) corresponding to a previously reported DAM^45^, and a reactive oligodendrocyte subtype (OLG-7, *C4b*^+^, *Serpina3n*^*+*^^46,47^). With the scATAC-seq dataset, we further confirmed its expansion (Fig. 3f and Extended Data Fig. 8b,c) and identified its associated TFs. For example, the OLG-7 associated TF, *Stat3* (Fig. 3e), plays a critical role in regulating inflammation and immunity in the brain^48^. We also performed a spatial transcriptomics experiment using adult and aged mouse brains. Strikingly, we detected a significant enrichment of the reactive oligodendrocyte-specific markers (for example, *C4b* and *Serpina3n)* around the subventricular zone (SVZ) (Fig. 3g,h), indicating an age-related activation of inflammation signaling around the adult neurogenesis niche.

**Extended Data Fig. 9:**
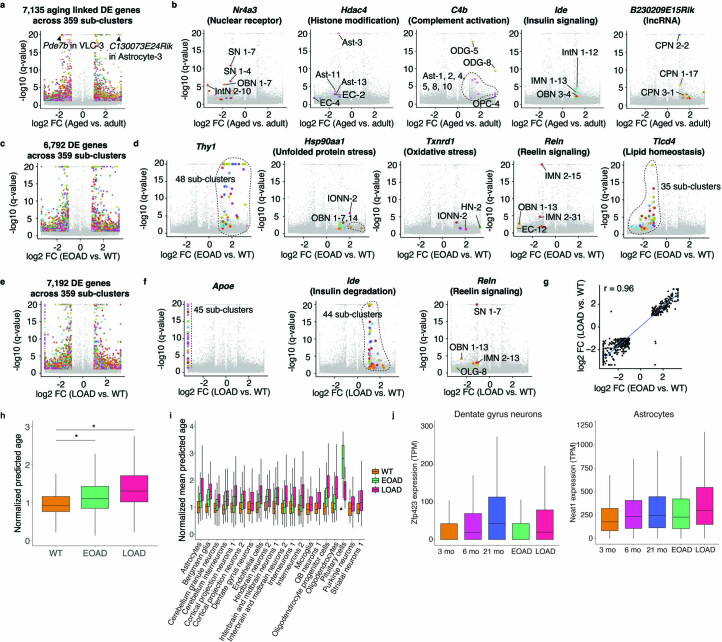
Identifying aging and AD pathogenesis-associated gene expression signatures. **a-f**. Volcano plots showing the differentially expressed (DE) genes between adult (6 months) and aged (21 months) mice (**a-b**), between WT and EOAD (**c-d**) and between WT and LOAD (**e-f**). Significantly changed genes are colored by the main cell type identity. Examples with concordant changes across subclusters are labeled with related biological pathways. **g**. Scatter plot shows the correlation of the log2-transformed fold changes of 559 DE genes significantly changed within the same subcluster in both AD models compared to WT. **h**. Box plot displays the predicted age of 3-month-old WT mice (n = 4745 pseudobulk cells) against AD models (EOAD: n = 11959; LOAD: n = 25152) across various cell types, normalized by the mean predicted age of the WT samples. Stars indicate p-value < 0.05 (WT vs EOAD: p = 7.6 × 10^-142^; WT vs LOAD: p = 0) using the two-sided Wilcoxon rank-sum test. **i**. Box plots displaying the predicted biological age of 3-month-old wild-type mice against AD models, showing cell types and conditions demonstrating significant differences (FDR adjusted p-value < 0.05, two-sided Wilcoxon rank-sum test,). Values are normalized by the mean predicted age of the WT samples. Number of pseudobulk cells per box plot (left to right): 179, 615, 1190, 37, 85, 179, 1819, 3272, 7875, 87, 220, 498, 493, 1701, 3057, 85, 359, 673, 62, 338, 65, 194, 424, 11, 30, 56, 605, 1096, 2732, 427, 1389, 2403, 77, 261, 483, 49, 158, 298, 63, 276, 50, 302, 59, 216, 432, 293, 880, 1837, 19, 3, 8, 35, 226, 118, 1178. **j**. Box plots showing *Zfp423* expression in dentate gyrus neurons (3 months = 301, 6 months = 204, 21 months = 334, EOAD = 192 and LOAD = 338 pseudobulk cells) and *Neat1* expression in astrocytes (3 months = 968, 6 months = 969, 21 months = 700, EOAD = 615 and LOAD = 1190 pseudobulk cells) across various conditions. Both genes, ranking among the top predictors of age in their respective cell types, exhibited increased expression in the 3-month-old AD models (*Zfp423* solely in the LOAD model, *Neat1* in both AD models). For all box plots, boxes indicate the median and interquartile range (IQR) with whiskers indicating 1.5X IQR.

We next explored the subtype manifestation of aging signatures by differentially expressed (DE) gene analysis. We identified 7,135 aging-associated signatures across 359 subclusters (Supplementary Table 9 and Extended Data Fig. 9a). Of the 580 genes significantly altered in multiple (≥3) subtypes, 241 showed consistent directions. For example, *Nr4a3* (genes involved in DNA repair^49^) was significantly decreased in aged neuron subtypes (striatal neurons, OB neurons, and interneurons). *Hdac4*, encoding a histone deacetylase^50^, decreased in aged astrocytes and ependymal cells. Insulin-degrading enzyme (IDE), involved in amyloid-beta clearance^51^, also increased in neuron subtypes. We also identified age-related changes in non-coding RNAs, many with high cell-type specificity (for example, *B230209E15Rik* in cortical projection neuron subtypes), but were not well characterized previously (Extended Data Fig. 9b).

### AD pathogenesis-associated gene signatures and cell subtypes

**Fig. 4:**
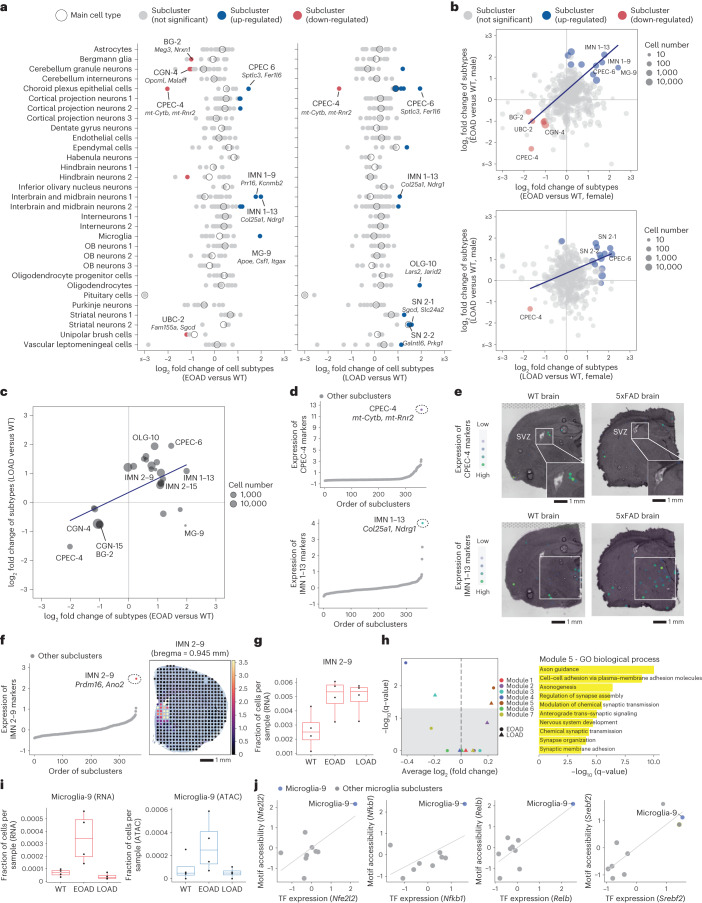
Identifying AD pathogenesis-associated cell subtypes. **a**, Dot plots showing the log-transformed fold changes of main cell types (circles) and subclusters (dots) comparing EOAD versus WT (left) and LOAD versus WT (right). Significantly changed subclusters were colored by the direction of changes. Representative subclusters were labeled along with top gene markers. **b**, Scatter plots showing the correlation of the log fold change of subclusters (top: EOAD versus WT; bottom: LOAD versus WT) between males and females. **c**, Scatter plot showing the correlation of the log fold change of subclusters in two AD models (both compared with the WT). Only subclusters with significant changes in at least one AD model are included. Black/gray reflects overlapping dots. **d**, Scatter plots showing the aggregated expression of gene markers of two cell subtypes (top: CPEC-4; bottom: the IMN 1–13) across all subclusters from *EasySci-RNA* data. **e**, Brain coronal sections showing the spatial expression of subtype-specific gene markers of two subtypes (top: CPEC-4; bottom: the IMN 1–13) in the WT and EOAD brains in 10x Visium data. **f**, Scatter plot showing the aggregated expression of gene markers of IMN 2–9 across all subclusters from EasySci-RNA data (left) and the mapping score per pixels of the IMN 2–9 subcluster cells on the bregma 0.945 mm coronal section highlighting the lateral septal nucleus region^28^ (right). **g**, Box plots showing the fraction of IMN 2–9 cells across conditions (WT: *n* = 4 mice, EOAD: *n* = 4 mice, LOAD: *n* = 4 mice) profiled by EasySci-RNA. **h**, GM expression differences in both AD models against WT control (left) and the top enriched Gene Ontology (GO) biological processes pathways of module 5 genes (right). **i**, Box plots showing the fraction of microglia-9 cells across conditions (WT: *n* = 4 mice, EOAD: *n* = 4 mice, LOAD: *n* = 4 mice) profiled by EasySci-RNA (left) or EasySci-ATAC (right). **j**, Scatter plot showing the correlated gene expression and motif accessibility of four TFs enriched in microglia-9, together with a linear regression line. Boxes in box plots indicate the median and IQR with whiskers indicating 1.5× IQR.

Through comparison of subcluster fractions in two AD models to age-matched WT controls (3 months old), we detected 16 and 14 significantly changed subclusters (FDR of 5%, at least twofold change) in the EOAD (5xFAD) model and LOAD (APOE*4/Trem2*R47H) model, respectively (Fig. 4a and Supplementary Tables 10 and11). Most significantly altered subtypes correlated between genders (Fig. 4b) and between the two AD models, even though they had distinct genetic perturbations in different cell types (Fig. 4c). For example, a rare choroid plexus epithelial cell subtype (CPEC-4) was strongly depleted (by more than twofold decrease) in both models. This cell type is marked by significant enrichment of multiple mitochondrial genes linked to neuroprotective factors against neurodegeneration (for example, *mt-Rnr2* (ref. ^52^)) or Tau protein levels in cerebrospinal fluid (*mt-Rnr1* and *mt-Nd5*^53^). Through spatial transcriptomics analysis, we verified its location around the SVZ and confirmed its depletion in the EOAD (5xFAD) model, suggesting mitochondrial dysfunction in choroid plexus epithelial cells plays a role in neurodegenerative diseases (Fig. 4d,e).

By contrast, another choroid plexus epithelial cell subtype (CPEC-6; marked by *Sptlc3*^[+54^, *Fer1l6*^+^) expanded in both AD models (over twofold increase) (Fig. 4b). A similar expansion was observed in a rare interbrain and midbrain neuron subtype (IMN 1–13, marked by *Col25a1*^+^*, Ndrg1*^+^) that expresses *Col25a1*, a membrane-associated collagen reported to promote intracellular amyloid formation in mouse models^55^ (Fig. 4c). Spatial transcriptomic analysis confirmed the up-regulation of IMN 1–13 specific gene markers in the thalamus region of the 5xFAD mouse brain (Fig. 4d,e), providing further validation of the AD-related neuron subtype change. Additionally, a septal nuclei neuron subtype IMN 2-9 (marked by *Prdm16* and *Ano2*) that significantly overexpress in both AD model GMs related to axonogenesis (for example, *Nrp1* and *Slit2*) and synaptogenesis (for example, *Ptprd* and *Nrxn1*) (ref. ^56^) was also significantly expanded in both AD models (Fig. 4f–h), aligning with the observed enlargement of the septal nuclei region several years before the onset of memory decline^57^.

Meanwhile, we observed a significant expansion of microglia subtype 9 (marked by *Apoe* and *Csf1*) in early-onset 5xFAD mice, aligning with previous reports^45^. This disease-associated microglia (DAM) also expanded in aged mice but was not evident in the late-onset APOE*4/Trem2*R47H model at 3 months of age (validated by both RNA and ATAC), potentially indicating a correlation with disease onset (Fig. 4i). We further investigated its DE genes (Extended Data Fig. 8d) and key TFs exhibiting consistency between cell-type-specific gene expression and motif accessibility (Fig. 4j). The enriched TFs were reported to be involved in microglia expansion during aging and AD^58–60^. Additionally, we quantified the enrichment of genetic variants linked to human traits^61^ and observed significant enrichment of AD heritability in microglia cells at both the main cell type level and particularly in the microglia-9 subtype, highlighting the role of DAM in AD pathogenesis (Extended Data Fig. 8e,f).

We identified subtype-specific manifestations of key AD-related molecular signatures. In the 5xFAD (EOAD) model, we found 6,792 subcluster-specific DE genes, whereas the APOE*4/Trem2*R47H (LOAD) model had 7,192 subcluster-specific DE genes (Extended Data Fig. 9c,f and Supplementary Tables 12 and 13). The *Apoe* gene was globally down-regulated in the APOE*4/Trem2*R47H mice, possibly due to the replacement of the *Apoe* gene with the human sequence. Many AD-associated gene signatures exhibited consistent changes across cellular subtypes, such as increased stress-related markers (for example, *Hsp90aa1* and *Txnrd1*) in neuron subtypes in the 5xFAD mice. The expression of *Reln*^62^ decreased in various cell types in both models, aligning with previous report of *Reln* depletion before the onset of amyloid-beta pathology in the human frontal cortex^63^. Other intriguing observations included the down-regulation of *Tlcd4*, a gene involved in lipid trafficking and metabolism^64^ in multiple subclusters in the 5xFAD mice. Interestingly, despite genetic differences and disease onsets in the two AD models, there were remarkably consistent alterations in cell-type-specific molecular profiles. We identified 559 subcluster-specific DE genes shared between both AD mutants, suggesting common molecular mechanisms between early- and late-onset AD models (Extended Data Fig. 9g). We also investigated the connection between aging and AD-associated changes using transcriptomic aging clocks^65^, revealing significantly accelerated biological aging in both AD models (Extended Data Fig. 9h). Although most cell types demonstrated accelerated aging-related molecular changes, specific cell types only exhibited these signs in LOAD (Extended Data Fig. 9i). This is further validated by consistent cell-type-specific changes of aging-associated gene signatures (for example, *Neat1* and *Zfp423*) in aged and AD models (Extended Data Fig. 9k).

### Detection of dysregulated gene signatures in human AD brains

**Fig. 5:**
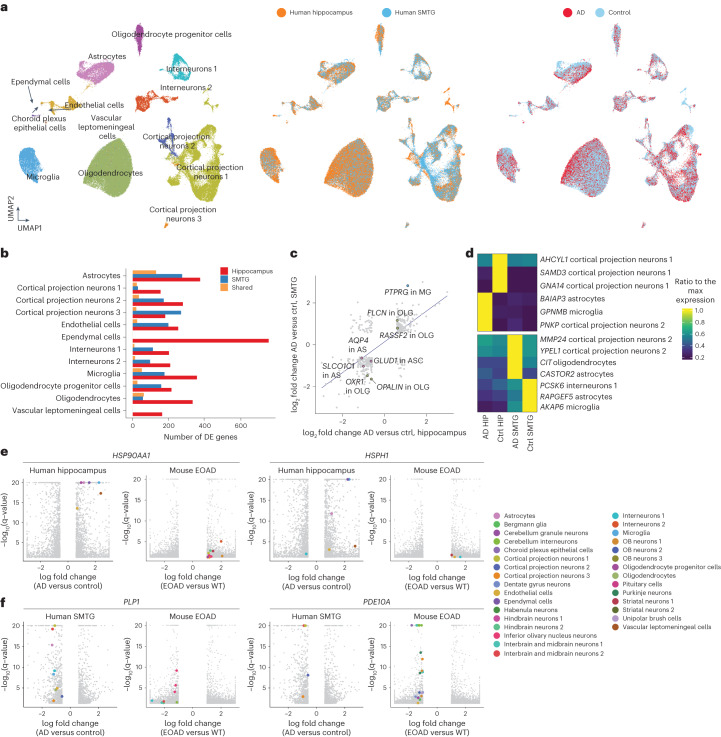
Identifying AD pathogenesis-associated gene expression signatures across regions and cell types in human brains. **a**, UMAP visualization of single-cell transcriptomes of all human brain cells, colored by main cell types (left), region (middle) and conditions (right). **b**, Bar plot showing the number of DE genes between AD and control samples in each cell type, colored by whether they are unique to each region or shared between two regions. Choroid plexus epithelial cells and vascular leptomeningeal cells were not included into the differential gene expression analysis in SMTG due to their low cell numbers. **c**. We detected 394 DE genes significantly changed within the same main cell type in both regions. The scatter plot shows the correlation of the log_2_-transformed fold changes of these 394 shared DE genes in the hippocampus (*x* axis) and in SMTG (*y* axis). Key genes are annotated and colored by their corresponding main cell types. **d**, Heatmaps showing examples of region-specific DE genes for the hippocampus (left) and SMTG (right). Gene expressions were quantified as TPM in the corresponding cell types in each group, and normalized to the maximum expression across groups. **e**,**f**, Volcano plots showing the examples of top DE genes between the AD and control samples across main cell types in human brains (**e**) or between EOAD and WT samples across cell subclusters in mouse brains (**f**). Highlighted genes are colored by the main cell-type identity.

**Extended Data Fig. 10:**
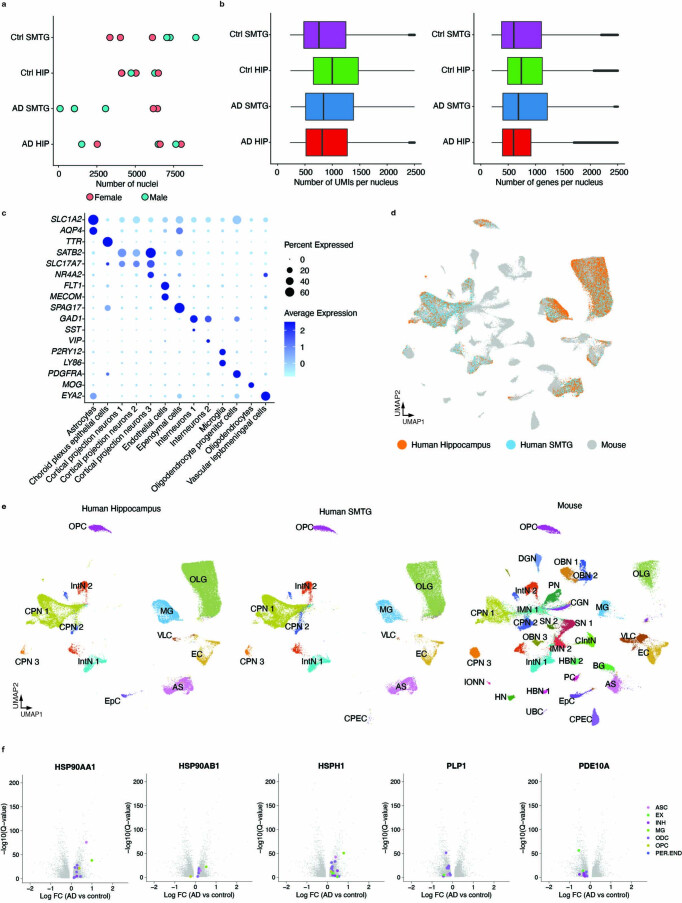
Quality control of the human brain dataset and conserved gene expression changes across mouse AD models and human AD samples from the prefrontal cortex. **a**. Scatter plot showing the number of single-cell transcriptomes profiled in each human sample in two regions, colored by sexes. **b**. Box plots showing the number of unique transcripts (left) and genes (right) detected per nucleus profiled in the human dataset (Control SMTG: n = 36,781 cells, Control HIP (hippocampus): n = 30,801 cells; AD SMTG: n = 17,864 cells, AD HIP: n = 32,794 cells). **c**. Dotplot showing the markers for the main cell types identified in the human dataset. **d**-**e**. UMAP plot showing the integration between human and mouse cells, colored by the dataset (**d**) and main cell types (**e**). AS, astrocytes; BG, Bergmann glia; CGN, cerebellum granule neurons; CIntN, cerebellum interneurons; CPEC, choroid plexus epithelial cells; CPN 1, cortical projection neurons 1; CPN 2, cortical projection neurons 2; CPN 3, cortical projection neurons 3; DGN, dentate gyrus neurons; EC, endothelial cells; EpC, ependymal cells; HN, habenula neurons; HBN 1, hindbrain neurons 1; HBN 2, hindbrain neurons 2; IONN, Inferior olivary nucleus neurons; IMN 1, interbrain and midbrain neurons 1; IMN 2, interbrain and midbrain neurons 2; IntN1, interneurons 1; IntN2, interneurons 2; MG, microglia; OBN 1, OB neurons 1; OBN 2, OB neurons 2; OBN 3, OB neurons 3; OPC, oligodendrocyte progenitor cells; OLG, oligodendrocytes; PC, pituitary cells; PN, purkinje cells; SN 1, striatal neurons 1; SN 2, striatal neurons 2; UBC, unipolar brush cells; VLC, vascular leptomeningeal cells. **f**. Volcano plots showing AD-associated gene expression changes from human prefrontal cortex samples^6^ colored by main cell types. These genes show consistent changes in multiple cell subclusters between mouse AD models, human hippocampus and SMTG samples, and human prefrontal cortex samples from the above-mentioned publication. ASC, astrocytes; EX, excitatory neurons; INH, inhibitory neurons; MG, microglia; ODG, oligodendrocytes; OPC, oligodendrocyte progenitor cells; PER.END, pericyte/endothelial cells.

To compare molecular signatures associated with AD pathogenesis in mouse models and human patients, we sequenced a total of 118,240 single-nuclei transcriptomes (a median of 5,585 nuclei per sample, with the sequencing depth of 13,850 raw reads and a median of 1,109 UMIs per nucleus; Extended Data Fig. 10a,b) from 24 human brain samples across two brain regions (hippocampus, superior and middle temporal gyrus (SMTG)), derived from six patients with AD and six age- and gender-matched controls (Supplementary Table 14). Thirteen main cell types were identified through integration analysis with the mouse dataset and validated by the specific expression of known markers (Fig. 5a and Extended Data Fig. 10c–e).

A total of 4,171 and 2,149 cell-type-specific DE genes were identified in the hippocampus and SMTG, respectively (Fig. 5b and Supplementary Table 15). Exactly 349 genes were significantly changed in the same cell type from two distinct regions, among which 332 were altered consistently (Fig. 5c). For example, oligodendrocytes in AD samples from both regions exhibited decreased expression of the oligodendrocyte terminal differentiation factor *OPALIN*^66^ and the oxidation stress protector *OXR1* (ref. ^67^). Concurrently, we observed an up-regulation of genes related to programmed cell death (for example, *FLCN* and *RASSF2*)^68,69^, suggesting an elevated stress in oligodendrocytes from AD brains. Other examples include the microglia-specific up-regulation of *PTPRG*^70^, and astrocyte-specific down-regulation of several transmembrane transporters (for example, *AQP4*) and neurotransmitter metabolism enzymes (for example, *GLUD1*)^71,72^.

Interestingly, some AD-associated gene signatures exhibited region-specific expression patterns. For example, *GPNMB*, encoding a transmembrane glycoprotein associated with microglia activation in AD brains^73^, showed increased expression in the microglia from the hippocampus but not from the SMTG. On the other hand, *MMP24*, encoding a member of the metalloproteinase family implicated in AD pathogenesis^74^, showed increased expression in cortical projection neurons unique within the SMTG (Fig. 5d). Notably, inhibition of MMP24 has been demonstrated to decrease amyloid-beta levels and promote cognitive functions in mouse models^75^, suggesting its potential role as a novel therapeutic target for AD.

Finally, we explored the human-mice relevance for AD-associated gene signatures and molecular pathways. Despite differences in the species and disease stages between the two datasets, several genes encoding heat shock proteins (for example, *HSP90AA1* and *HSPH1*) were up-regulated across multiple cell types in both species (Fig. 5e). The elevated chaperon system potentially reduces the formation of toxic oligomeric assemblies in AD brains^76^, further validating the dysfunction of proteostasis as a molecular marker of AD^77^. Meanwhile, we identified down-regulated genes in both human and mice. One of the examples, *PLP1*, was reported as a subtype-specific driver gene contributing to AD pathogenesis^78^. Another gene, *PDE10A*, plays a key role in promoting neuronal survival, with its reduction detected in our datasets and multiple neurodegenerative diseases (for example, Huntington’s disease^79^ and Parkinson’s disease^80^) (Fig. 5f). Importantly, the above-mentioned trends were readily validated by another single-cell dataset investigating AD in the human prefrontal cortex^6^ (Extended Data Fig. 10f). In summary, the human-mice relevance analysis identified species-conserved genetic programs associated with AD pathogenesis.

## Discussion

In this study, we introduced EasySci, a cost-effective technical framework for individual laboratories to generate gene expression and chromatin accessibility profiles from millions of single cells. We used EasySci to analyze 1.5 million single-cell transcriptomes with full gene body coverage and 380,000 chromatin accessibility profiles across mammalian brains of different ages and genotypes. The datasets enable the identification of over 300 cellular subtypes throughout the brain, including highly rare cell types representing less than 0.01% of the total brain cell population. Furthermore, we discovered region-specific effects attributable to aging and AD and examined the manifestation of molecular signatures associated with aging and AD on a cell-type-specific basis.

As highlighted by our subcluster level analysis, the effects of aging and AD on the global brain cell population are profoundly cell-type specific. Although most brain cell types stay relatively stable under various conditions, we identified over 50 cell subtypes exhibiting over twofold change in brains affected by aging and AD models. Many of these cell subtypes were rare and overlooked in conventional single-cell analysis. For example, the aging brain is characterized by the depletion of both rare neuronal progenitor cells and differentiating oligodendrocytes, associated with the enrichment of a *C4b*^*+*^ *Serpina3n*^*+*^ reactive oligodendrocyte subtype surrounding the SVZ, suggesting a potential interplay between oligodendrocytes, localized inflammatory signals and the stem cell niche.

The lack of reliable mouse models remains a big challenge in studying late-onset AD. The novel APOE*4/Trem2*R47H model aims to overcome this limitation by introducing two of the strongest late-onset AD-associated mutations^81^. We found consistent molecular and cellular population dynamics between the well-established 5xFAD and the novel APOE*4/Trem2*R47H model. For example, we observed shared subtypes that were depleted (for example, *mt-Cytb*^*+*^ *mt-Rnr2*^*+*^ choroid plexus epithelial cell) or enriched (for example, *Col25a1*^*+*^ *Ndrg1*^*+*^ interbrain and midbrain neuron) in both early- and late-onset AD mutant brains. Meanwhile, differences were also observed between the two AD models, as expected by the different onset times. The absence of an increase in the DAM population in the LOAD model may be due to its lack of amyloid deposition^82^ or by genetic perturbations, as both *Trem2* and *Apoe* play a role in the activation of this cell population^45^.

In addition, we investigated AD-associated gene signatures in human brains by profiling over 100,000 single-nucleus transcriptomes derived from 24 human brain samples from control and AD patients, across two distinct anatomical locations. Although most AD-associated gene dynamics are profoundly cell-type and region specific, we identified dysregulated genetic signatures that are conserved between different locations in the human brains. Moreover, integrating the human and mouse brain datasets further revealed molecular pathways shared between human AD patients and mouse AD models, which suggests that the mouse AD model can serve as a model system to investigate the function and regulation of these conserved features associated with AD or neuronal dysfunction.

Of note, there are several inherent limitations of the study. First, the analysis covers only around 2% of the total mouse brain population (estimated at approximately 100 million cells), which means extremely rare cell subtypes may still be overlooked. Additionally, our relatively shallow sequencing depth might hinder the detection of lowly expressed transcripts or minor aging-related cellular state changes. Nevertheless, the validity of our key biological findings is reinforced by the consistent results across different genders (male versus female), genotypes (EOAD versus LOAD), and orthogonal approaches (such as comparisons between single-cell transcriptome, chromatin accessibility or spatial transcriptomics). This lends significant credence to our discoveries, even when considering the limitations of the study.

In summary, we have showcased the power of highly scalable single-cell genomics to delve into the dynamics of rare cell types, uncovering novel subtypes associated with aging and disease. Though our focus was on brain tissues, the strategic approach could be readily extended to systematically explore cellular states across an entire organism. Such exploration could illuminate the rare vulnerable cell populations to aging and diseases, opening up pathways to develop targeted therapeutic strategies.

## Methods

### Animals

C57BL/6 WT mouse brains at 3 months (*n* = 4), 6 months (*n* = 4) and 21 months (*n* = 4) were collected in this study. Two AD models at 3 months old from the same C57BL/6 background were added, including an early-onset model (5xFAD, JAX stock #034840) that overexpresses mutant human amyloid-beta precursor protein with the Swedish (K670N, M671L), Florida (I716V) and London (V717I) familial AD (FAD) mutations and human presenilin 1 harboring two FAD mutations, M146L and L286V. Brain-specific overexpression is achieved by neural-specific elements of the mouse *Thy1* promoter^16^. The second, late-onset AD model (APOE*4/Trem2*R47H, JAX stock #028709) in this study carries two of the highest risk factor mutations of LOAD^81^, including a humanized *APOE* knock-in allele, where exons 2 and 3 and most of exon 4 of the mouse gene were replaced by the human ortholog including exons 2, 3, 4 and some part of the 3’ UTR. Furthermore, a knock-in missense point mutation in the mouse *Trem2* gene was also introduced, consisting of an R47H mutation, along with two other silent mutations. Two male and two female mice are included in each condition. Mice were housed socially. All animal procedures were in accordance with institutional, state, and government regulations and approved under institutional animal care and use committee protocols 21049 and 20047.

### EasySci-RNA library preparation

Detailed step-by-step EasySci-RNA protocol is included as Supplementary Protocol 1.

### Human brain sample

Twenty-four post-mortem human brain samples across two regions (hippocampus and SMTG) and twelve individuals, including six controls and six patients with AD, ranging from 70 to 94 years of age, were collected from the University of Kentucky AD Center Tissue Bank. Each included participant who donated samples for this study signed a relevant consent form (including consent for unrestricted sharing of clinical, pathological and genetic information for dementia research) that was approved by the UK Internal Review Board (UK IRB #44009).

### Computational procedures for processing *EasySc*i-RNA libraries

A custom computational pipeline was developed to process the raw fastq files from the EasySci libraries. Similar to our previous studies^10,11^, the barcodes of each read pair were extracted. Both adaptor and barcode sequences were trimmed from the reads. Second, an extra trimming step is implemented using Trim Galore^85^ with default settings to remove the poly(A) sequences and the low-quality base calls from the cDNA. Afterward, the paired-end sequences were aligned to the genome with the STAR aligner^86^, and the PCR duplicates were removed. Finally, the reads are split into SAM files per cell, and the gene expression is counted using a custom script. The reads from the same cell originating from the short dT and the random hexamer RT primers were counted as independent cells. During the gene counting step, we assigned reads to genes if the aligned coordinates overlapped with the gene locations on the genome. If a read was ambiguous between genes and derived from the short dT RT primer, we assigned the read to the gene with the closest 3’ end; otherwise, the reads were labeled as ambiguous and not counted. If no gene was found during this step, we then searched for candidate genes 1,000 bp upstream of the read or genes on the opposite strand. Reads without any overlapped genes were discarded. Similar strategy was used for generating an exon count matrix across cells.

To compare the performance of EasySci-RNA with the commercial 10x Chromium system, we subsampled ~3,800 raw reads/cell from one randomly selected PCR batch of our large-scale mouse brain experiment, a 10x v2 Chromium dataset^83^, a 10x v3 dataset (https://www.10xgenomics.com/resources/datasets/5k-adult-mouse-brain-nuclei-isolated-with-chromium-nuclei-isolation-kit-3-1-standard) and a SPLiT-seq dataset^33^. After the subsampling, the EasySci data were processed with the custom computational pipeline, whereas the 10x Chromium data were processed with 10x Genomics’ Cell Ranger software^87^. We removed low-quality cells (unassigned reads >30%, UMIs >20,000 and genes <200) and selected the top 1,000 highest-quality cells from the 10x Chromium dataset^83^ and a deeply sequenced EasySci-RNA library profiling adult mouse brains. Subsequently, we subsampled these cells to different sequencing depths and quantified the unique transcripts/genes detected per cells. Based on this comparison, we recommend a sequencing depth of no less than 5,000 raw reads per nucleus to ensure adequate coverage and detection of a substantial number of unique molecules.

### Cell clustering and annotation analysis

After gene counting, we kept the cells with reads identified by both RT primers. We then merged the reads from the same cells. Low-quality cells were removed based on one of the following criteria: (i) the percentage of unassigned reads > 30%, (ii) the number of UMIs >20,000 and (iii) the detected number of genes <200. We then used the Scrublet^88^ to identify and remove potential doublets. To identify distinct clusters of cells, we subjected the 1,469,111 single-cell gene expression profiles to UMAP visualization and Louvain clustering, similar to our previous study^10^. We then co-embedded our data with the published datasets^2,89,90^ through Seurat^91^, and clusters were annotated based on overlapped cell types. The annotations were manually verified and refined based on marker genes. DE genes across cell types were identified with the differentialGeneTest() function of Monocle 2 (ref. ^92^). To identify cell-type-specific gene markers, we selected genes that were DE across different cell types (FDR of 5%, likelihood), with over twofold expression difference between first and second-ranked cell types and TPM >50 in the first-ranked cell types.

### Cell subclustering analysis

We selected each main cell type and applied PCA (combined matrix including the 30 principal components derived from the gene-level expression matrix and the first 10 principal components derived from the exon-level expression matrix), UMAP and Louvain clustering similarly to the major cluster analysis. We then merged subclusters that were not readily distinguishable in the UMAP space similar as described before^10^. DE genes and exons across cell types were identified with the differentialGeneTest() function of Monocle 2 (ref. ^92^). To identify subcluster-specific DE genes associated with aging or AD models, we sampled a maximum of 5,000 cells per condition for downstream DE gene analysis using the differentialGeneTest function of the Monocle 2 (ref. ^92^). The sex of the animals was included as a covariate to reduce sex-specific batch effects.

To detect cellular fraction changes at the subtype level across various conditions, we first generated a cell count matrix by computing the number of cells from every subcluster in each RT well profiled by EasySci-RNA. Each RT well was regarded as a replicate comprising cells from a specific mouse individual. Of note, we repeated the same analysis using the number of cells from each subcluster in each mouse individual (instead of RT well) and the result is highly consistent. We then applied the likelihood ratio test to identify significantly changed subclusters between different conditions, with the differentialGeneTest() function of Monocle 2^92^. Subclusters were removed if they had <20 cells in either the male or female samples. The fold change was calculated by normalizing the number of cells in a cluster by the total number of cells in the corresponding condition, then dividing the normalized values in the case and control conditions after adding a small number (10^−5^) to reduce the effect of the very small clusters. In addition, we considered subclusters to change significantly only if there was over twofold change between conditions and the q-value was less than 0.05.

### Integration analysis with external datasets and to locate the spatial distributions of main cell types and subtypes

To annotate the spatial locations of main cell types, we integrated the EasySci-RNA data with publicly available 10x Visium spatial transcriptomics datasets (https://www.10xgenomics.com/resources/datasets/mouse-brain-section-coronal-1-standard-1-0-0, https://www.10xgenomics.com/resources/datasets/mouse-brain-serial-section-1-sagittal-anterior-1-standard-1-0-0; https://www.10xgenomics.com/resources/datasets/mouse-brain-serial-section-1-sagittal-posterior-1-standard-1-0-0) through a NNLS approach: we first aggregated cell-type-specific UMI counts, normalized by the library size, multiplied by 100,000 and log-transformed after adding a pseudocount. A similar procedure was applied to calculate the normalized gene expression in each spatial spot captured in the 10x Visium dataset. We then applied NNLS regression to predict the gene expression of each spatial spot in 10x Visium data using the gene expression of all cell types recovered in Easy-RNA data, similar to our previous study^10^. The same approach^10^ was applied to integrate our EasySci-RNA dataset with a large single-cell dataset featuring highly detailed cell type annotations^2^ for identification of shared cellular states in two datasets.

To spatially map EasySci cell subtypes, we first aggregated ~50 single-cell transcriptomes identified by k-means clustering of cells in the UMAP space of subclustering analysis. We then integrated the EasySci-RNA data with the above 10x Visium spatial transcriptomics datasets and a published spatial dataset^28^, using cell2location^27^ following the default settings. To establish the corresponding regions of EasySci subclusters, we utilized the regional annotation of the spatial pixels and manually reviewed the anatomical regions of the top 10 pixels with the highest mapping score. To remove low-quality spatial mappings, only mapping scores above 1 were considered.

### GM analysis

We performed GM analysis to identify the molecular programs underlying different cell types in the brain. First, we aggregated the gene expression across all subclusters. The aggregated gene count matrix was then normalized by the library size and then log-transformed. Genes were removed if they exhibited low expression (less than 1 in all subclusters) or low variance of expression (that is, the gene expression fold change between the maximum expressed subcluster and the median expression across subclusters is less than 5). The filtered matrix was used as input for UMAP visualization^19^ (metric = ‘cosine’, min_dist = 0.01, n_neighbors = 30). We then clustered genes based on their 2D UMAP coordinates through densityClust package (rho = 1, delta = 1)^93^.

### EasySci-ATAC library preparation and sequencing

The detailed protocol for EasySci-ATAC library preparation is included in Supplementary Protocol 2.

### Data processing for EasySci-ATAC

After sequencing, base calls were converted to fastq format and demultiplexed using Illumina’s bcl2fastq/v2.19.0.316 tolerating one mismatched base in barcodes (edit distance <2). Downstream sequence processing was similar to sci-ATAC-seq^94^. To compare the performance of EasySci-ATAC with other methods, we extracted reads containing barcodes from cells passing quality control (3,636 cells from one PCR well of the EasySci-ATAC library, 8067 nuclei from the 10x-ATACv2 library and 5,494 nuclei from the sci-ATAC-seq library^15^). We normalized for sequencing depth differences by subsampling reads from the 10X-ATACv2 and sci-ATAC-seq library, resulting on average 6,360 raw reads per cell across all three libraries. We processed the data through the same computation pipeline described above. Peak calling was performed on each dataset separately with these parameters:–nomodel–extsize 200–shift -100 -q 0.05. For peak counting, a union peak set was generated by merging the peaks called from three datasets. Cells were determined to be accessible at a given peak if a read from a cell overlapped with the peak. The peak-count matrix was generated by a custom python script with the HTseq package^95^.

### Cell filtering, clustering and annotation for EasySci-ATAC

We used SnapATAC2/v1.99.99.3^96,97^ to preprocess the EasySci-ATAC dataset. Cells with <1500 fragments and <2 TSS Enrichment were discarded. Potential doublet cells and doublet-derived subclusters were detected using an iterative clustering strategy^10^ modified to suit for scATAC-seq data. We then used a deep-learning-based framework scJoint^21^ to annotate main ATAC-seq cell types by using the *EasySci-RNA* dataset as a reference. First, we subsampled 5,000 cells from each main cell type of the *EasySci-RNA* dataset, and selected genes detected in more than 10 cells. Then, the gene count matrix and cell type labels of *EasySci-RNA*, along with the gene activity matrix of *EasySci-ATAC* were input into the scJoint pipeline with default parameters. Jointed embedding layers calculated from scJoint were used for UMAP visualizations using python package umap/v0.5.3 (ref. ^19^). Louvain clusters were identified using the Seurat function FindNeighbors() and FindClusters() based on the UMAP coordinates. Cells were assigned to the prediction label with the highest abundance within each louvain cluster. Clusters with low purities (that is, <80% cells were from the highest abundant cell type) were removed. Finally, to validate the integration-based annotations, we selected DE genes identified from the RNA-seq data with the following criteria: fold change between the maximum and the second maximum expressed cell type >1.5, q-value < 0.05, TPM >20 in the maximum RNA group and reads per million >50 in the maximum ATAC group. The top 10 DE genes ranked by fold change were selected using RNA-seq data for each cell type. If there were less than 10 genes passing the cutoff, we selected the top genes ranked by the fold change between the maximum expressed cell type and the mean expression of other cell types. We then calculated the aggregated gene count and gene body accessibility for each cell type. Subcluster level integrations were similar to the main cluster level integrations.

### Differential accessible peak analysis

Nonduplicate ATAC-seq reads of cells from each main cell type were aggregated and peaks were called on each group separately with these parameters:–nomodel–extsize 200–shift -100 -q 0.05 using MACS2/v2.1.1 (ref. ^98^). To correct for differences in read depth or the number of nuclei per cell type, we converted MACS2 peak scores (−log_10_(q-value)) to ‘score per million’^99^ and filtered peaks by choosing a score-per-million cutoff of 1.3. Peak summits were extended by 250 bp on either side and then merged with bedtools/v2.30.0. Cells were determined to be accessible at a given peak if a read from a cell overlapped with the peak. The peak-count matrix was generated by a custom python script with the HTseq package^95^.

We used R package Signac/v1.7.0 (ref. ^100^) to perform the dimension reduction analysis using the peak-count matrix. We subsampled 5,000 cells from each main cell type and performed TF-IDF normalization using RunTFIDF(), followed by singular value decomposition using RunSVD() and retained the 2nd to 30th dimensions for UMAP visualizations using RunUMAP(). Differentially accessible peaks across cell types were identified using monocle 2 (ref. ^92^) with the differentialGeneTest() function. 5,000 cells were subsampled from each cell type for this analysis. Peaks detected in less than 50 cells were filtered out. We selected peaks that were differentially accessible across cell types by the following criteria: 5% FDR (likelihood ratio test), and with TPM >20 in the target cell type.

### Transcription factor motif analysis

We used ChromVar/v1.16.0 (ref. ^101^) to asess the TF motif accessibility using cisBP motif sets curated by chromVARmotifs/v0.2.0 (ref. ^101,102^). We subsampled 5,000 cells from each main cell type, and calculated the motif deviation score for each single cell using the Signac wrapper RunChromVAR(). The motif deviation scores of each single cell were rescaled to (0, 10) using R function rescale() and then aggregated for each cell type. In addition, we also aggregated the gene expression of each TF in each cell type. We then computed the Pearson correlations between the aggregated motif matrix and aggregated TF expression matrix after scaling across all main cell types. TF analysis at the subcluster level was performed similarly with modifications. For each cell type of interest, we selected peaks detected in more than 20 cells and only kept cells with more than 500 reads in peaks. Peaks were resized to 500 bp (±250 bp around the center) and motif occurrences were identified using matchMotifs() function from motifmatchr/v1.16.0 (ref. ^103^). The motif deviation matrix was calculated using the ChromVar function computeDeviations(). Then, the motif deviation scores were rescaled to (0, 10) and aggregated per subcluster. Pearson correlation was calculated between the aggregated motif activity and aggregated TF expression across subclusters after scaling (subclusters with <20 cells were excluded).

### LDSC analysis

The LDSC computational pipeline was modified from Cusanovich et al.^15^ and based on the LDSC software^104^ (https://github.com/bulik/ldsc). Specifically, to integrate human and mouse data, we first used the UCSC utility liftOver^105^ to lift all GWAS SNPs to the mouse genome. We then took the set of differentially accessible peaks across main clusters and across microglia subclusters, and annotated each SNP according to whether or not it overlapped one of these peaks. We then followed the recommended workflow for running LDSC using HapMap SNPs^106^, precomputed files corresponding to 1000 genomes phase 3, excluding the MHC region to generate an LDSC model for each chromosome and peak set. Only main cell types or subclusters containing DEpeaks in every chromosome are included in the following analysis.

To calculate enrichments based on each model, we first regenerated the baseline model (version 1.1) provided from the LDSC website and used this as the reference for enrichment calculation. Results for all trait/cluster pairs were gathered into a single file. *P* values were calculated from z-scores assigned to coefficients reported by ldsc.py and coefficients were divided by the average per-SNP heritability for traits associated with a given test. Tests were corrected for multiple hypothesis testing using the Benjamini-Hochberg method and only tests with a q-value of 0.05 or lower were considered significant.

### *Cis*-regulatory elements linkage analysis

We first constructed pseudo-cells by aggregating the RNA-seq and ATAC-seq profiles of the same subclusters. Aggregated count matrices were normalized to TPM and log-transformed after adding one pseudocount. We only retained genes and peaks with TPM value greater than 10 in the maximum expressed pseudo-cells. Then, for each gene, we calculated the Pearson correlation coefficient (PCC) between its gene expression and the chromatin accessibility of its nearby accessible sites (±500 kb from the TSS) across aggregated subclusters. To define a threshold at PCC score, we also generated a set of background pairs by permuting the subcluster ID of the ATAC-seq matrix and with an empirically defined significance threshold of FDR < 0.01, to select significant positively correlated *cis*-reculatory element-gene pairs. We only keep the top linked gene with the highest PCC for each peak and distal peaks overlapping with the promoters for other genes were filtered out.

### Spatial gene expression profiling of mouse brains

Spatial gene expression analysis experimental protocol was followed according to Visium Spatial Gene Expression User Guide (catalog no. CG000160), Visium Spatial Tissue Optimization User Guide (catalog no. CG000238 Rev A, 10x Genomics) and Visium Spatial Gene Expression User Guide (catalog no. CG000239 Rev A, 10x Genomics).

### Transcriptomic aging clock analysis

A ridge regression model was employed to predict the ln(age) of pseudobulk cells (on average 15 cells merged) utilizing 80% of the pseudobulk cells from 3, 6, and 21-month-old mice. Predicted ages were subsequently calculated for the remaining 20% of WT mice and the entirety of the AD models. Individual models were crafted for each cell type.

### Clustering, annotation and differential analysis for human brain samples

A digital gene expression matrix was constructed from the raw sequencing data as described before. To identify distinct clusters of cells corresponding to different cell types in the human brain samples, we co-embedded the human cells from both regions with our mouse brain dataset (up to 5,000 cells randomly sampled from each of 31 cell types), and clusters were annotated based on overlapped cell types. The annotations were manually verified and refined based on marker genes. Following on, the hippocampus and SMTG human dataset were integrated together to construct the same low-dimensional space with only human cells.

DE genes between AD and control samples for each cell type in each region were identified using Monocle 2 (ref. ^107,108^) with the differentialGeneTest() function. Main cell types with less than 50 cells were excluded from the analysis (that is, choroid plexus epithelial cells and vascular leptomeningeal cells in the SMTG). DE genes were filtered based on the following cutoffs: q-value < 0.05, with fold change (FC) > 1.5 between the maximum and second expressed condition, and with TPM >50 in the highest expressed condition. To further validate human-mouse shared gene expression changes, we used a recently published AD single-cell dataset from the human prefrontal cortex^6^.

### Statistics and reproducibility

Statistical analyses are detailed in figure legends (Fig. 1 and Extended Data Fig. 9) and were performed using R software (version 4.0.1). The number of cells or pseudobulk cells used for the comparisons are detailed in the figure legends and the number of replicates are detailed in Methods. For spatial integration analysis in Fig. 1k,l, Fig. 3g,h, Fig. 4e and Extended Data Fig. 5h,i, each spatial transcriptomic datum includes one section of the experiment.

### Reporting summary

Further information on research design is available in the Nature Portfolio Reporting Summary linked to this article.

## Online content

Any methods, additional references, Nature Portfolio reporting summaries, source data, extended data, supplementary information, acknowledgements, peer review information; details of author contributions and competing interests; and statements of data and code availability are available at 10.1038/s41588-023-01572-y.

### Supplementary information

Supplementary Information Supplementary Protocols 1 and 2.

Reporting Summary

Supplementary Table 1 1) Summary of key optimizations of EasySci-RNA compared to published sciRNA-seq3. 2) Differentially expressed genes across main cell types. 3) Differentially accessible sites across main cell types. 4) Differentially expressed exons across subclusters within each main cell type. 5) Validation of EasySci-RNA subclusters using integration analysis. 6) Gene module analysis results. 7) Differentially abundant subclusters between adult and young samples. 8) Differentially abundant subclusters between aged and adult samples. 9) Differentially expressed genes between aged and adult for all subclusters. 10) Differentially abundant subclusters between wild type and EOAD model. 11) Differentially abundant subclusters between wild type and LOAD model. 12) Differentially expressed genes between wild type and EOAD model (5xFAD) for all subclusters. 13) Differentially expressed genes between wild type and LOAD model (APOE*4/Trem2*R47H) for all subclusters. 14) Metadata for human samples included in this study. 15) Differentially expressed genes between control and AD human brain samples for main cell types in each region.16) Short dT reverse transcription primer sequences. 17) RandomN reverse transcription primer sequences. 18)Ligation primer sequences. 19) EasySci-RNA P7 primer sequences. 20) Primers for preparing barcoded Tn5 plates for EasySci-ATAC. 21) EasySci-ATAC P7 primer sequences.

## Data Availability

All relevant data generated in this study are deposited to public repositories and are publicly released. Raw and processed data of single-cell RNA-seq/ATAC-seq profiling were deposited at the NCBI Gene Expression Omnibus (https://www.ncbi.nlm.nih.gov/geo/query/acc.cgi?acc= GSE212606).

## Code availability

Customized computational scripts of processing EasySci data were deposited in Zenodo^109^ (10.5281/zenodo.8395492) and GitHub (https://github.com/JunyueCaoLab/EasySci).
