# Supplementary material for: A global view of aging and Alzheimer’s pathogenesis-associated cell population dynamics and molecular signatures in human and mouse brains
Source: Nat Genet. 2023 Nov 30;55(12):2104–16. doi: 10.1038/s41588-023-01572-y (PMC10703679; doi:10.1038/s41588-023-01572-y)
Supplement: Supplementary file 1 — Supplementary Protocols 1 and 2. [file 41588_2023_1572_MOESM1_ESM.pdf]

nature genetics

Article https://doi.org/10.1038/s41588-023-01572-y

A global view of aging and Alzheimer's pathogenesis-associated cell population dynamics and molecular signatures in human and mouse brains

In the format provided by the authors and unedited

Supplementary information for: A global view of aging and Alzheimer's pathogenesis-associated cell population dynamics and molecular signatures in human and mouse brains.

Supplementary Protocols

- Supplementary protocol 1: EasySci-RNA protocol
- Supplementary protocol 2: EasySci-ATAC protocol

References

Supplementary Protocol 1: EasySci-RNA protocol

Protocol workflow

- Buffer Preparation (Steps 1-12)
- Ligation Primer Annealing (Steps 13-16)
- Tn5 loading (Step 17)
- Nuclei Extraction (Steps 18-26, ~2.5 hours)
- Nuclei Wash (Steps 27-28, ~15-30 minutes,)
- Nuclei Counting (Step 29)
- Reverse Transcription (Steps 30-33, ~1-2.5 hours)
- Pool/Centrifuge/Resuspend/Redistribute (Steps 34-35, 15 minutes)
- Ligation (Steps 36-40, ~2 hours)
- Pool/Centrifuge/Resuspend/Redistribute/Quantify (Steps 41-45, 30 minutes)
- Second-Strand Synthesis (Steps 46-48, ~1.25 hours)
- 0.8x Ampure Beads Purification (Steps 49-55, ~1 hour)
- Tagmentation (Steps 56-57, ~10 minutes)
- SDS Treatment (Steps 58-61, ~1.5 hours)
- PCR (Step 62, ~45 minutes)
- Library Purification (Steps 63-74, ~1 hour)

To validate the experimental setup, it is recommended to start with a species-mixing experiment. We normally start with a mixture of human (HEK 293T) and mouse (NIH/3T3) cells. A good run normally yields single-cell transcriptomes with over 5000 UMIs (with over 20,000 sequencing reads) per cell and >90% purity.

Required Equipments

- Bioruptor Sonication Device
- Hemocytometers (Neubauer Improved, Bulldog Bio VWR #102966-632)
- Centrifuge (Eppendorf 5702 RH)
- DynaMag-96 Side Skirted Magnet (Invitrogen, 12027) / DynaMag-96 Side Magnet (Invitrogen, 12331D)
- 12-tube Magnetic Separation Rack (NEB, S1509S)
- Eppendorf Mastercycler (4x)
- Freezer (-20C, -80C) and Refrigerator (4C)
- Gel Imager
- Ice Buckets
- Microscope
- Multi-channel Pipettes (2-20μL, 20-200μL) (Rainin Instruments)
- Pipettors
- 96 well Pipetting System
- Liquid nitrogen tank for sample storage
- FreezeCell Cell Freezing Container (GeneSeeSci, catalog number: 27-802) Eppendorf ThermoMixer C (5382000023) OR Fisherbrand Nutating Mixer (88861043)

Primer Sequences used

All primer sequences including RT/Ligation/PCR primers are attached as a separate excel file (EasySci-RNA_primer_sequences.xlsx). All primers are ordered from IDT with standard desalting.

Materials used

- Nuclease free water (Ambion, AM 9937)
- 10cm cell culture dish (Genesee, 25-202)
- 6cm cell culture dish (Genesee, 25-260)
- OEMTOOLS 25181 Razor Blades, 100 Pack (VWR, 55411-0055)
- Ward's 40um Sterile Cell Strainer (VWR, 470236-276)
- PluriStrainer Mini 40um (PluriSelect 43-10040-70)
- PluriStrainer Mini 20um (PluriSelect 43-10020-70)
- PluriStrainer Mini 5um (PluriSelect 43-10005-70)
- BD New STERILE , Sealed , 5 ML Syringes Only LUER Lock TIP, No Needle, Disposable (VWR, BD309646)
- Pierce 16% Formaldehyde, Methanol Free (Thermofisher, 28906)
- SUPERase In RNase Inhibitor 20 U/uL (Thermo Fisher Scientific, AM2696)
- BSA 20 mg/ml (NEB, B9000S)
- 1M Tris-HCl (pH 7.5) (Thermo Fisher Scientific, 15567027)
- 5M NaCl (Thermo Fisher Scientific, AM9759)
- 1M MgCl2 (Thermo Fisher Scientific, AM9530G)
- TE Buffer (IDTE, 11-05-01-05)
- Dimethylformamide, 99.8% (Fisher Scientific, AC327175000)
- Dimethyl Sulfoxide (VWR, 97063-136)
- Nuclei Isolation Kit: Nuclei EZ Prep (Millipore Sigma, NUC101-1KT)
- Diethyl Pyrocarbonate (DEPC) (VWR, 97062-652)
- PBS, 1X (Genesee, 25-507)
- Triton X-100 for molecular biology (Sigma Aldrich, 93443-100ML)
- 10mM dNTP (Thermo Fisher Scientific, R0192)
- 192 indexed shortdT primers (100uM, 5′-/5Phos/ACGACGCTCTTCCGATCTNNNNNNNN[10bp barcode]TTTTTTTTTTTTTTTT-3′, where “N” is any base; IDT)
- 192 indexed randomN primers (100uM, 5'-/5Phos/ACGACGCTCTTCCGATCTNNNNNNNN[10bp barcode]NNNNNN-3', where "N" is any base; IDT)
- Maxima H Minus Reverse Transcriptase with Buffer (ThermoFisher, EP0753)
- T4 DNA Ligase (NEB, M0202L)
- EDTA 0.5M Solution (VWR, 97062-656)
- 384 indexed ligation primers (100uM, 5’-AATGATACGGCGACCACCGAGATCTACAC[10bp barcode]ACACTCTTTCCCTAC-3’; IDT)
- Adapter Primer (100uM, 5'-A*G*A*T*C*G*G*A*A*G*A*G*C*G*T*C*G*T*G*T*A*G*G*G*A*A*A*G*A*G*T*G*T*/3ddC/, where '*' represents phosphorothioate bonds between nucleotides and '/3ddC/' represents a dideoxycytidine modification; IDT)
- Elution buffer (Qiagen, 19086)
- NEBNext® Ultra II Non-Directional RNA Second Strand Synthesis Module (NEB, E6111L)
- Tn5 enzyme (in-house produced following Hennig et al. 2018)
- DNA binding buffer (Zymo Research, D4004-1-L)
- AMPure XP beads (Beckman Coulter, A63882)
- SDS, 20% Solution, RNase Free (ThermoFisher AM9820)
- Tween 20 (Millipore Sigma, P9416-100ML)
- Ethanol (Sigma Aldrich, 459844-4L)
- Universal P5 primer (10 µM, 5′-AATGATACGGCGACCACCGAGATCTACAC-3′; IDT)
- 384 indexed P7 primers (10 µM, 5′-CAAGCAGAAGACGGCATACGAGAT[i7]GTCTCGTGGGCTCGG-3′; IDT)
- NEBNext High-Fidelity 2X PCR Master Mix (NEB, M0541L)
- Qubit dsDNA HS kit (Invitrogen, Q32854)
- Qubit tubes (Invitrogen, Q32856)

- E-Gel EX Agarose Gel, 2% (ThermoFisher, G402002)
- E-Gel 50bp DNA Ladder (ThermoFisher, 10488099)
- Falcon Tubes, 15 ml (VWR Scientific, 21008-936)
- Falcon Tubes, 50 ml (VWR Scientific, 21008-940)
- Green pack LTS 200ul filter tips (GP-L200F) (Rainin Instrument, 17002428)
- Pipette Tips RT LTS 20uL FL 960A/10 (Rainin, 30389226)
- Pipette Tips RT LTS 200uL F 960/10 (Rainin, 30389239)
- Pipette Tips RT LTS 200uL FLW 960A/10 (Rainin, 30389241)
- 4-Chip Disposable Hemocytometers, Neubauer Improved, Bulldog Bio (VWR, 102966-632)
- DNA LoBind Tube 1.5 ml, PCR clean (Eppendorf North America, 22431021)
- 1.0mL Self-Standing Cryovial (GeneSeeSci, catalog number: 24-200P)
- LoBind clear, 96-well PCR Plate (Eppendorf North America, 30129512)
- 0.2mL 8-Strip Tubes with Individual Caps (PCR Tubes) (Genesee, 27-125U)
- Reagent reservoirs (Fisher Scientific, 07-200-127)
- Falcon® 5mL Round Bottom w/ Cell Strainer (Fisher Scientific, 352235)
- eXTReme FoilSeal Film (Genesee, 12-156)
- eXTReme Clear Sealing Film (Genesee, 12-157)

Buffer Preparation

1. **500mL Nuclei Buffer (Stored in 4 °C)**
   10mM Tris-HCl, pH 7.5; 10mM NaCl; 3mM $MgCl_2$ in nuclease free water:

**Reagent**	**Stock concentration**	**Final concentration**	**Volume (ml)**
Tris-HCl (pH 7.5)	1 M	10 mM	5
NaCl	5 M	10 mM	1
MgCl2	1 M	3 mM	1.5
Nuclease-free water	NA	NA	492.5
Final volume			500

   Filter the buffer through a 0.22uM filter and store the buffer in 4 °C for up to 1 year.

2. **20 mL 10% (volume) Triton-X-100 in nuclease-free water (stored in 4 °C)**
   Add 2 mL Triton X-100 to 18 mL nuclease-free water. Mix the solution by pipetting up and down 20 times. The mix can be stored in 4 °C for up to 1 year.

3. **EZ Lysis Buffer + 0.1% RNase Inhibitor (Made fresh each time, stored on ice, 2 mL per tissue sample)**
   EZ lysis buffer with 0.1% (volume) SUPERase In RNase Inhibitor . For each sample, combine 2 mL EZ lysis buffer and 2 µL SUPERase In RNase Inhibitor.

4. **EZ Lysis Buffer + 1% DEPC (Made fresh each time, stored on ice, DEPC added just before lysis step, 1 mL per tissue sample)**
   EZ Lysis buffer with 1% (volume) DEPC. For each sample, combine 990 µL EZ lysis buffer and 10 µL DEPC

5. **Nuclear Suspension Buffer (NSB) (Made fresh each time, stored on ice)**
   Nuclei Buffer with 1% SUPERase In RNase Inhibitor and 1% BSA: For every 1 mL NSB needed, combine 980 µL Nuclei Buffer, 10 µL SUPERase In RNase Inhibitor, and 10 µL BSA.

6. **Nuclear Suspension Buffer + 10% DMSO (NSB + 10% DMSO) (Made fresh each time, 100 µL needed per sample aliquot, stored on ice)**
   For every 1 mL needed, add 900 µL Nuclear Buffer and 100 µL DMSO.

7. **Nuclear Suspension Buffer + 0.1% Triton-X-100 (NSB + Triton) (Made fresh each time, 750 µL needed per sample, stored on ice)**
   For every 1 mL needed, add 990 µL Nuclei Buffer and 10 µL 10% Triton-X-100.

8. **Nuclear Buffer + 1% BSA + 0.1% Triton-X-100 (NBB) (Made fresh each time, ~8mL needed, store on ice)**
   Add 7.84 mL Nuclei Buffer, 80 µL BSA, and 80 µL 10% Triton-X-100.

9. **0.1% Formaldehyde in PBS (Made fresh each time, 1 mL needed per sample, store on ice)**
   For every 1 mL solution needed, add 1 mL PBS and 6.25 µL 16% Formaldehyde (Using 1mL glass vial of 16% formaldehyde: open and use a fresh tube of formaldehyde each time)

10. **2x Tagmentation Buffer (Stored in -20 °C)**
    Prepare 200 mL of Tagmentation Buffer (filtered):

    - 1M Tris HCl (pH 7.5): 4mL
    - 1M $MgCl_2$: 2mL
    - DMF: 40mL
    - H_2O: 154mL

    Aliquot the solution into 15 mL or 1.5 mL tubes for storage at -20 °C.

11. **1% SDS (Stored at room temperature)**
    Mix 1 mL 10% SDS and 9 mL H_2O

12. **10% Tween-20 (Stored in 4 °C)**
    Mix 1 mL Tween-20 and 9 mL H_2O, let sit for 10 minutes before mixing again. Repeat until the solution is homogeneous.

Ligation Primer Loading (1 hour)

13. Resuspend and dissolve the Ligation Adaptor Primer Oligo to 100 µM concentration in TE Buffer

14. In each well of an empty 96-well plate, add 5 µL of 100 µM dissolved Ligation Adaptor Primer and 5 µL 100 µM Barcoded Ligation Primers.

15. Anneal the adaptor and ligation primers together by running the following thermocycler program:

    - 95 °C for 2 minutes
    - Cool to 20 °C at a rate of -1 °C per minute
    - Hold at 4 °C

    The final annealed concentration will be 50 µM.

16. Dilute the primers to 3.125 µM by adding 150 µL of EB buffer. The resulting product is in stable, double-stranded form and can be stored at 4 °C or frozen. In 4 °C, the annealed primers should be stable for roughly three months and are suitable for short-term testing experiments.

Tn5 Loading (1 hour)

17. Protocol Derived from (Hennig et al. 2018), the purified Tn5 protein was also generated following this publication. The Tn5 loading procedure is listed below:

    - First mix 150 µL of 100 µM Tn5-ME-B oligo (5'-GTCTCGTGGGCTCGGAGATGTGTATAAGAGACAG-3', in TE buffer) with 150 µL of 100 µM Tn5-ME-rev oligo (-/5'Phos/CTGTCTCTTATACACATCT-3', in TE buffer) reaching a final concentration of 50 µM
    - Split the mixture into aliquots and perform the following thermocycler conditions: 95 °C for 5 minutes, slowly cooled to 65 °C (0.1C/sec or 2%), 65 °C for 5 minutes, slowly cooled to 4 °C (0.1C/sec or 2%).
    - Further dilute the mixture to 35 µM by mixing 10 µL of the oligo mixture with 4.28 µL of TE buffer.
    - Combine 1 µL of the Tn5 enzyme at 4 mg/mL with 19 µL of Tn5 Dilution Buffer (25 mM Tris pH 7.5, 800 mM NaCl, 0.1 mM EDTA, 1 mM DTT and 50% glycerol) and 2 µL of the 35 µM Tn5-ME-B/Tn5-MErev oligo mixture.
    - Place this solution on a thermomixer at 23 °C for 30 minutes and dilute with 22 µL of glycerol and store at -20 °C for future usage.

    Alternatively, use unloaded Tn5 from Diagenode (Catalog. C01070010-10).

Nuclei Extraction (~2.5 hours for 6 samples)

18. Cool centrifuge to 4 °C - make sure to use a bucket centrifuge for all centrifuging steps unless otherwise stated, as normal centrifuges may have difficulty making a neat pellet at the bottom of the tube, which is necessary to maximize nuclear recovery.
    In a 6 cm dish on ice, cut each tissue section (0.1 g - 0.5 g) into small pieces (< 1 mm^3) using a razor blade and 1 mL PBS with 10 µL DEPC added. Transfer the tissue and solution into a 1.5 mL tube and spin for 5 minutes at 200g at 4 °C.

    NOTES:
    - Make sure to add DEPC just before performing lysis, as DEPC has a short half-life in aqueous solutions
    - Perform this step in a fume hood, as chopping tissue in a DEPC solution may be toxic
    - For larger tissue samples, splitting into multiple 1.5 mL tubes is advised to make pipetting the samples easier
    - Ideally, you don't want the tissue sections to thaw until the sections are being cut in the DEPC-PBS solution. To prevent thawing, have a separate container filled with dry ice to place the sections that are currently not being minced with the razor blade
    - Generally, we work with a maximum of six tissue sections at one time - you can theoretically process more at the same time, but it may be difficult to manage

19. Dump Supernatant and add 1 mL ice-cold EZ lysis buffer + 1% DEPC to the tissue for nuclei extraction. Pipet the tissue up and down with a 1 mL pipet tip 10 times (cut the top of 1 mL pipet tip if needed for easier pipetting). Incubate on ice for 5 minutes.

NOTES:

- Make sure to add DEPC just before performing lysis, as DEPC has a short half-life in aqueous solutions and will degrade if not added immediately before lysis
- From this point on, use 1 mL pipet tips or wide bore tips when working with nuclei to avoid stress on nuclei

20. Filter tissue with a 40 µm cell strainer into a 6 cm dish and grind tissue on the strainer using a 5 mL syringe plunger. Add 500 µL EZ Lysis Buffer + 0.1% RNase Inhibitor and continue grinding tissue on the strainer. Move solution into a 1.5 mL microcentrifuge tube.

    NOTES:

    - It is not necessary to push the whole tissue through the filter! Use your own discretion in deciding when to stop grinding the tissue through the filter, but make sure not to tear through the filter!

21. Pellet the nuclei by centrifuging for 5 minutes, 500g at 4 °C. Dump supernatant. Resuspend each tube in 500 µL EZ Lysis Buffer + 0.1% RNase Inhibitor by pipetting up and down three times.

22. Pellet the nuclei by centrifuging for 5 minutes, 500g at 4 °C. Dump supernatant.

23. **Fixation:** Take each tube and add 1 mL of ice-cold 0.1% Formaldehyde suspended in PBS. Start a 10-minute timer immediately after formaldehyde is added. Mix up and down to resuspend the pellet. For multiple samples, add 1 mL directly to the top of tubes without changing tips and without touching the tubes; start timer once the formaldehyde is added to all tubes. Once done, go back and pipet up and down the solution in each sample to resuspend the pellet, making sure to switch tips for each sample.

    NOTES:

    - Perform this step in a fume hood as formaldehyde is toxic

24. Pellet the nuclei immediately afterward by centrifuging for 3 minutes, 500g at 4 °C. Dump supernatant in a chemical waste container. Resuspend each tube in 500 µL EZ Lysis Buffer + 0.1% RNase Inhibitor by pipetting up and down three times.

25. Pellet the nuclei by centrifuging for 5 minutes, 500g at 4 °C. Dump supernatant. Resuspend each tube in 500 µL EZ Lysis Buffer + 0.1% RNase Inhibitor by pipetting up and down three times.

26. PERFORM THIS STEP IF YOU WISH TO STORE NUCLEI FOR LATER USE - OTHERWISE, SKIP TO THE SECOND PART OF THE NEXT STEP:
    Pellet the nuclei by centrifuging for 5 minutes, 500g at 4 °C. Resuspend each tube in 100-500 µL NSB + 10% DMSO and split into 100 µL aliquots. Slow freeze in a -80 °C freezer and keep for storage. Optimally, use specialized slow-freezing chambers with 1.0 mL Self-Standing Cryovials (FreezeCell Cell Freezing Container, GeneSeeSci, catalog number: 27-802) (1.0 mL Self-Standing Cryovial, GeneSeeSci, catalog number: 24-200P) **(STOP POINT)**.

Nuclei Wash (~15-30 minutes for 6-30 samples)

27. 1) PERFORM BELOW IF YOU ARE WORKING WITH PREVIOUSLY FROZEN, STORED NUCLEI:
    Thaw cells for 30 seconds in a 37 °C water bath. Add 400 µL NSB + Triton to each sample to resuspend pellets, and then sonicate for 12 seconds at low power. After the sonication, filter nuclei through a 20 µm filter. Wash the filter with an additional 250 µL NSB + Triton and then pellet the nuclei for 5 minutes, 500g at 4 °C.

2) PERFORM BELOW IF YOU ARE DIRECTLY CONTINUING FROM NUCLEI EXTRACTION:
Add 500 µL NSB + Triton to each sample to resuspend pellets, and then sonicate for 12 seconds at low power. After, filter nuclei through a 20 µm filter. Wash the filter with an additional 250 µL NSB + Triton and then pellet the nuclei for 5 minutes, 500g at 4 °C.

28. Resuspend the pellet in 100 µL of NSB.

Nuclei Counting

29. Count the nuclei concentration for each sample.

Optimally, use a buffer with DAPI and a fluorescent microscope to distinguish between actual nuclei and debris.
Dissolve 10 mg DAPI in 2 ml of deionized water (dH2O) with a final concentration of 5 mg/ml. Split the DAPI solution into multiple tubes (100 ul per tube).
Take out one tube (100 ul, 5 mg/ml DAPI), add 1.9 ml deionized water (dH2O). Split the diluted DAPI solution into multiple tubes (100 ul per tube, 0.25 mg/ml DAPI).
Store the DAPI solution in the -20 °C freezer.

Make the DAPI counting solution: in 500 µL of Nuclei Buffer, add 0.5 µL - 1µL of 0.25mg/mL DAPI solution.
Take 1 µL of the sample and combine it with 9 uL of the counting solution. Mix the solution and take 6 µL to dispense into a hemocytometer for counting the concentration of nuclei.

Reverse Transcription (~1 - 2.5 hours depending on number of samples)

30. For each well of 2 x 96 well plates, add a maximum of 20,000 nuclei in 4 µL of NSB; also add 0.5 µL of 10 mM dNTP.

NOTES:
- Nuclei generally distributed into PCR strips and then distributed into wells - make sure not to pipet up and down to avoid introducing extra stress to the nuclei
- It is recommended to use wide bore multichannel tips for mixing the nuclei

31. Add 1 µL of 50 µM short-dT primer and 1 µL of 50 µM randomN primer into each well using multi-channel pipettes or 96-well liquid handler. Incubate plates at 55 °C for 5 minutes. Immediately place plates on ice afterward.

32. Prepare the reverse transcription reaction mix by combining:

- 5X Maxima Buffer: 420µL
- Maxima Reverse Transcriptase: 105µL
- SUPERase In RNase Inhibitor: 105µL
- Nuclease Free H2O: 105µL

Add 3.5µL to each well for each of the plates; pipet up and down only once

33. Start the reverse transcription with the following thermocycler program:

- 4 °C for 2 minutes
- 10 °C for 2 minutes
- 20 °C for 2 minutes

- 30 °C for 2 minutes
- 40 °C for 2 minutes
- 50 °C for 2 minutes
- 55 °C for 15 minutes

Pool/Centrifuge/Resuspend/Redistribute (15 minutes)

34. Add 10 µL NBB into each well, pool solution, and move solution into a 15 mL tube. Centrifuge the tube for 3 minutes, 1000g at 4 °C.

35. Use a pipet to aspirate supernatant. Resuspend nuclei in 1 mL NBB and then transfer the nuclei into a 1.5 mL microcentrifuge tube. Centrifuge the tube for 3 minutes, 1000g at 4 °C to pellet the nuclei.

Ligation (1 hour)

36. Dump the supernatant. Resuspend the cells in 950 µL NBB. Distribute the nuclei into four PCR plates, with 2.5 µL of the solution going into each well.

37. To each well, add 1 µL of the appropriate DNA ligation primer/adaptor complex (3.125 µM).

38. Create a mixture of:

    - 210 µL 10X T4 Ligation Buffer
    - 21 µL SUPERase In RNase Inhibitor
    - 210 µL T4 DNA Ligase
    - 189 µL Nuclease Free Water

    Add 1.5 µL of the mixture to each of the PCR plate wells.

39. Incubate plates for 30 minutes at room temperature with gentle shaking (300 rpm with Thermomixer, 50 rpm on Fisherbrand Nutating Mixer).

40. Add 1 µL EDTA (18 mM diluted in nuclease free water) into each well and pool all solution into a 15 mL tube.

Pool/Centrifuge/Resuspend/Redistribute/Quantify (30 minutes)

41. Centrifuge the tube for 3 minutes, 1000g at 4 °C. Pipet out the supernatant.

42. Resuspend the nuclei in 1 mL NBB. Transfer the cells into a microcentrifuge tube. Centrifuge the tube for 3 minutes, 1000g at 4 °C. Dump the supernatant.

43. Resuspend the nuclei in 500 µL NBB. Filter the nuclei using a 40 µM filter and then wash the filter with an additional 250 µL NBB. Centrifuge the tube for 3 minutes, 1000g at 4 °C. Dump the supernatant.

44. Resuspend the nuclei in 500 µL NBB for nuclei counting - it is recommended to use a fluorescent microscope with a solution with DAPI to distinguish nuclei from debris.

45. Distribute the nuclei into a 96 well plate with 10,000 nuclei per well, suspended in 4 µL total volume (final concentration = 2,500 nuclei/µL).

NOTES:

- You can directly freeze and store cells at this point, but it is recommended to proceed directly to second-strand synthesis as dsDNA should be more stable in storage compared to ssDNA
- If you choose to freeze the plate, it is okay to place it directly in -80 °C freezer without flash-freezing
- You can also store nuclei directly into PCR strips if you don't need to profile a whole plate of cells

Second-Strand Synthesis (1 hour 15 minutes)

46. Thaw Second-Strand Synthesis buffer in room temperature.

47. Prepare Second-Strand Synthesis mix: for each well, add ⅔ μL Second-Strand Synthesis buffer + ⅓ μL Second-Strand Synthesis Enzyme Mix.

48. Perform Second-Strand Synthesis in Thermocycler: incubate samples at 16 °C for one hour. **(STOP POINT).**

0.8x Ampure Beads Purification (~1 hour for one plate)

49. Take one plate of prepared cells after Second-Strand Synthesis and add 5 μL DNA binding buffer to each well, mix, and let the resulting solution sit for 5 minutes at room temperature.

    NOTES:
    - You can also perform this protocol with PCR strips if you do not need to profile a whole plate

50. Add 8 μL ampure beads to each well, mix well via pipetting, and let the resulting solution sit for 5 minutes at room temperature.

51. Place the solution on a magnetic rack and let the solution sit for 5 minutes.

52. Remove the resulting supernatant and add 50 μL of 80% ethanol (do not mix up and down). Remove the ethanol.

53. Wash one more time with 50 μL of 80% ethanol (do not mix up and down). Remove the ethanol, centrifuge the pellet down, place the plate on the magnetic rack, and remove the remaining residual ethanol.

54. Take the plate off of the magnetic rack and elute the beads in 7.6 μL of elution buffer. Incubate the solution for three minutes at room temperature.

55. Place the plate back on the magnetic rack and let the plate sit for three minutes at room temperature. Aspirate 6.6 μL of solution without touching the magnetic beads and transfer the solution into a new plate.

Tagmentation (10 minutes)

56. Prepare a mixture of 1:100 Tagmentase:Tagmentation Buffer mix. Add 6.6 μL of the mix to each well and pipet up and down to mix.

57. Incubate plate in the thermocycler at 55 °C for 5 minutes. Place on ice immediately following the reaction.

SDS Treatment (45 minutes)

58. For each well, add a mixture of:

    - 0.4 µL 1% SDS
    - 0.4 µL BSA
    - 2 µL 10 µM Universal P5 Primer

59. Incubate the plate at 55 °C for 15 minutes. Place the plate on ice immediately following the reaction.

60. Add 2 µL 10% Tween-20 to each well.

61. Add 2 µL Indexed P7 primer to each well. Centrifuge the plate after this step.

PCR (45 minutes)

62. Add 20 µL NEBNext Master Mix into each well and pipet up and down. Place samples into a thermocycler and run the following reaction:

    - 72 °C for 5 minutes
    - 98 °C for 30 seconds
    - 12-15 cycles of 98 °C for 10 seconds, 66 °C for 30 seconds, 72 °C for 30 seconds
    - 72 °C for 5 minutes

    NOTES:
    - It may be helpful to run a qPCR to determine the optimal number of cycles for amplification
    - You can store the resulting PCR products in -20 °C **(STOP POINT)**.

Library Purification (1 hour)

63. Pool all the wells together. Take 200 µL of the PCR product and perform a 0.8x ampure beads purification: start with adding 160 µL beads to the 200 µL of solution. Mix the solution via vortexing and let the resulting solution sit at room temperature for 5 minutes.

64. Place the solution on a magnetic rack and let the solution sit for 5 minutes until the beads are clearly separately from the solution.

65. Aspirate and remove the solution, making sure not to touch the beads. Add 1 mL of 80% ethanol to rinse beads and then remove the ethanol.

66. Add 1 mL of 80% ethanol for a second wash and then remove the ethanol.

67. Mix the bead with 105 µL of elution buffer followed by vortexing. Let the solution sit at room temperature for 3 minutes.

68. Place the solution on the magnetic rack after a brief centrifuge. Let the solution sit at room temperature for 3 minutes.

69. Transfer 100 µL of the solution into a new tube.

70. Quantify the library concentration and visualize the library via electrophoresis (performed using a

Qubit and a 2% Agarose E-Gel). An example library is shown below:

71. Sequence the library on the Novaseq Platform (Read1:100 bp, Read2: 100 bp, Index 1: 10 bp, Index 2: 10 bp).

Supplementary Protocol 2: EasySci-ATAC protocol

Protocol workflow

- Buffer Preparation (Steps 1-6)
- Ligation Primer Loading (Step 7-10)
- Tn5 loading (Step 11)
- Nuclei Extraction (Step 12-18)
- Nuclei Wash (Steps 19-21)
- Nuclei Counting (Step 22-23)
- Tagmentation (Steps 24-28)
- Pool/Centrifuge/Resuspend/Redistribute (Steps 29-34)
- Ligation (Steps 35-40)
- Pool/Centrifuge/Resuspend/Redistribute/Quantify (Steps 41-46)
- Proteinase K treatment (Step 47-50)
- PCR (Step 51-55)
- Sequencing Library Purification (Steps 56-61)

To validate the experimental setup, it is recommended to start with a species-mixing experiment. We normally start with a mixture of human (HEK 293T) and mouse (NIH/3T3) cells. A good run normally yields single-cell chromatin accessibility with 3000~4000 unique fragments (with over 10,000 sequencing reads) per cell and >90% purity.

Primer Sequences used

All primer sequences including Tn5 oligos/Ligation/PCR primers are attached as a separate excel file (EasySci-ATAC_primer_sequences.xlsx). All primers are ordered from IDT. Most are with standard desalting, except Tn5 oligos and universal P5 primers which we use HPLC purifications.

Required Equipments

- Hemocytometers (Neubauer Improved, Bulldog Bio VWR #102966-632)
- Centrifuge (Eppendorf 5702 RH)
- Eppendorf Mastercycler (4x)
- Freezer (-20C, -80C) and Refrigerator (4C)
- Gel Imager
- Ice Buckets
- Microscope
- Multi-channel Pipettes (2-20µL, 20-200µL) (Rainin Instruments)
- Pipettors
- Liquid nitrogen tank for sample storage
- FreezeCell Cell Freezing Container (GeneSeeSci, catalog number: 27-802) Eppendorf ThermoMixer C (5382000023) OR Fisherbrand Nutating Mixer (88861043)]

Materials used

- 1 M Tris-HCl, pH 7.5 (VWR, 97062-936)
- 5 M NaCl (VWR, 97062-858)

- 1 M MgCl2 (VWR, 97062-848)
- Tween-20 (Sigma, P9416)
- cOmplete™, EDTA-free Protease Inhibitor Cocktail (Sigma, 11873580001)
- IGEPAL CA-630 (VWR, IC0219859650)
- Dimethylformamide (Fisher, AC327175000)
- 0.5 M EDTA (VWR, 97062-656)
- Sperimidine (Sigma, S3256-1g)
- DMSO (VWR, 97063-136)
- T4 DNA Ligase (NEB, M0202L)
- EB buffer (Qiagen, 19086)
- TE Buffer (IDTE, 11-05-01-05)
- 10% SDS (VWR, E719-100ML)
- Proteinase K (Sigma, 3115828001)
- Tween-20 (Sigma, P9416)
- NEBNext® High-Fidelity 2X PCR Master MixNEB (NEB, M0541L)
- DNA Clean & Concentrator kit (Zymoresearch, D4014)
- Zymoclean Gel DNA Recovery Kit (Zymoresearch, D4007)
- 6-cm Cell culture dishes(Genesee, 25-260)
- Razor Blades (VWR, 100491-872)
- 1.5 mL Eppendorf™ DNA LoBind Tubes (Eppendorf, 22431021)
- 40 µm cell strainers (VWR, 470236-276)
- 5 mL Syringes (Fisher, 309603)
- pluriStrainer Mini 5 µm filter (Pluriselect, 43-10005-70)
- pluriStrainer Mini 40 µm filter (Pluriselect, 43-10040-70)
- 96-well plates (Genesee, 24-302)
- 2% E-Gel™ EX Agarose Gels (Invitrogen, G402022)
- E-Gel™ 50 bp DNA Ladder (Invitrogen, 10488099)
- Tn5 MEr oligos: /5Phos/CTGTCTCTTATACACATCT
- 12 indexed N5 oligos: (100 µM, /5Phos/ACGACGCTCTTCCGATCT[6bp-barcode]AGATGTGTATAAGAGACAG; IDT, HPLC purification)
- 32 indexed N7 oligos: (100 µM, CGTGTGCTCTTCCGATCT[6bp-barcode]AGATGTGTATAAGAGACAG; IDT, HPLC purification)
- Ligation Adapter Primer (100 µM, AGATCGGAAGAGCGTCGTGTAGGGAAAGAGTGT; IDT)
- 384 indexed ligation primers (100 µM, 5'-AATGATACGGCGACCACCGAGATCTACAC[10bp barcode]ACACTCTTTCCCTAC-3'; IDT)
- Universal P5 primer (10 µM, 5′-AATGATACGGCGACCACCGAGATCTACAC-3′; IDT, HPLC purification)
- 96 indexed P7 primers (10 µM, 5′-CAAGCAGAAGACGGCATACGAGAT[10bp-i7-barcode]GTGACTGGAGTTCAGACGTGTGCTCTTCCGATCT-3′; IDT)

Buffer Preparation

1. **500 mL Nuclei Buffer (NB, Stored in 4 °C)**
   10 mM Tris-HCl, pH 7.5; 10 mM NaCl; 3 mM $MgCl_2$ in nuclease free water:

Reagent	Stock concentration	Final concentration	Volume (ml)
Tris-HCl (pH 7.5)	1 M	10 mM	5
NaCl	5 M	10 mM	1
$MgCl_2$	1 M	3 mM	1.5
Nuclease-free water	NA	NA	492.5
Final volume			500

Filter the buffer through a 0.22 μm filter and store the buffer in 4C for up to 1 year.

2. **Nuclei Isolation Buffer (NIB)**
Nuclei buffer with 0.1% (volume) Tween-20 and 1X Protease Inhibitor Cocktail. To make a 25X Protease Inhibitor solution, dissolve 1 tablet of cOmplete™, EDTA-free Protease Inhibitor Cocktail into 2 mL nuclease free water.

3. **Nuclei Isolation Buffer + 0.1% IGEPAL-630**
Nuclei Isolation Buffer with 0.1% (volume) IGEPAL-630. For each sample, combine 1980 μL Nuclei Isolation Buffer and 20 μL 10% IGEPAL-630.

4. **2X Tagmentation Buffer (Stored in -20 °C)**

Prepare 200 mL of Tagmentation Buffer (filtered):

- 1M Tris HCl (pH 7.5): 4mL
- 1M $MgCl_2$: 2mL
- DMF: 40mL
- H_2O: 154mL

Aliquot the solution into 15 mL or 1.5 mL tubes for storage at -20 °C.

5. **2X Stop Buffer (Prepared fresh everytime, ~1.5 mL needed for one 96-well plate)**

Reagent	Stock Con.	Volume
EDTA	40 mM	400μL
Spermidine		0.78μL

Nuclease free water		4.6 mL

6. **Tn5 storage buffer**

   50 mM Tris-HCl, pH 7.45; 800 mM NaCl; 0.2 mM EDTA; 2 mM DTT; 10% glycerol.

   Filter the buffer through a 0.22 μm filter, aliquot into 15mL/1.5 mL tubes, and store the buffer in -20 °C.

Reagent	Stock concentration	Final concentration	Volume (ml)
Tris-HCl (pH 7.5)	1 M	50 mM	5
NaCl	5 M	800 mM	16
EDTA	40 mM	0.2 mM	0.5
DTT	500 mM	2 mM	0.4
Glycerol		10% (volume)	10
Nuclease-free water			68.1
Final Volume			100

Ligation Primer Loading (1 hour)

7. Resuspend and dissolve the Ligation Adaptor Primer Oligo to 100 μM in TE Buffer.

8. In each well of an empty 96-well plate, add 5 μL of 100 μM dissolved Ligation Adaptor Primer and 5 μL 100 μM Barcoded Ligation Primers - make sure to add the Barcoded Ligation Primers to their correct wells.

9. Anneal the adaptor and ligation primers together by running the following thermocycler program:

   - 95 °C for 2 minutes
   - Cool to 20 °C at a rate of -1 °C per minute
   - Hold at 4 °C

   The final annealed concentration will be 50 μM.

10. Dilute the primers to 3.3 μM by adding 140 μL of TE buffer. The resulting product is in stable, double-stranded form and can be stored at 4 °C or frozen. In 4 °C, the annealed primers should be

stable for roughly three months and are suitable for short-term testing experiments.

Tn5 Loading (2 hours)

11. Protocol Derived from Hennig et al. 2018, Large-Scale Low-Cost NGS Library Preparation Using a Robust Tn5 Purification and Tagmentation Protocol - purified Tn5 protein is also generated following this publication. The Tn5 loading procedure is listed below. Volumes are calculated to make 4 96-well barcoded Tn5 plates.

    1) For each N5 barcoded oligo, mix the following:
        - 11.52 µL 100 µM N5 barcoded oligo
        - 11.52 µL 100 µM MEr oligo
        - 48.96 µL Tn5 Storage buffer

    2) For each N7 barcoded oligos, mix the following:
        - 5.76 µL 100 µM N7 barcoded oligo
        - 5.76 µL 100 µM MEr oligo
        - 24.48 µL Tn5 Storage buffer

    3) Run the following PCR program in a thermocycler for the annealing of the oligonucleotides:
        - 95°C 5 min --> slowly cool down to 65 °C (0.1°C/sec or 2%)
        - 65°C 5 min --> slowly cool down to 4 °C (0.1°C/sec or 2%)
        - 4°C

    4) Plate indexed oligos in 96-well plate:
        - Mix 2.5 µL of N5/MEr (16 µM) and 2.5 µL of N7/MEr (16 µM) in each well.
        - Result: 5 µl of 8 µM Tn5 oligo mix.

    5) Prepare glycerol Tn5 stock:
        - Thaw 600 µL Tn5 extract on ice (4mg/ml original concentration)
        - Prepare 1800 µL glycerol buffer: 600ul Tn5 Storage buffer + 1200 ul 100% glycerol (800 mM NaCl) + 7.2 µL 500 mM DTT (final DTT concentration at 2 mM).
        - Add the thawed Tn5 to the glycerol buffer and mix by gentle rotation at 4 °C.
        - Once it is mostly mixed together, use a pipet to make it completely homogeneous. and store at -20 °C until needed.

    6) Load indexed Tn5:
        - Add 5.5 µL of 8 µM Tn5 stock (1 mg/ml) to each well, mix by pipetting gently.
        - Incubate at room temperature for 30 minutes with gentle shaking at 300 RPM.
        - Centrifuge the plates and store at -20 °C until needed.

Tissue preparation and nuclei isolations (~2 hours for 4 samples)

11. Cool centrifuge to 4 °C - make sure to use a bucket centrifuge for all centrifuging steps unless otherwise stated, as normal centrifuges may have difficulty making a neat pellet at the bottom of the tube, which is necessary to maximize nuclear recovery. In a 6-cm dish on ice, cut each tissue

section (0.1 g - 0.5 g) into small pieces (< 1 mm^3) using a razor blade (pre-chilled) in 1 mL PBS on ice. Transfer the tissue and solution into a 1.5 mL tube and spin for 5 minutes at 200g at 4 °C.

NOTES:

- Do not process too many samples at the same time to reduce over-lysis.
- Do not let the samples stand on ice for too long, start the nuclei extraction immediately after taking them out of the freezer.

12. Dump Supernatant and add 1 mL ice-cold NIB + 0.1% IGEPAL CA-630 to the tissue for nuclei extraction. Pipet the tissue up and down with a 1 mL pipet tip 10 times (cut the top of 1 mL pipet tip if needed for easier pipetting). Incubate on ice for 5 minutes.

    NOTES:

    - From this point on, use 1 mL pipet tips or wide bore tips when working with nuclei to avoid stress on nuclei.
13. Filter tissue with a 40 µm cell strainer into a 6 cm dish and grind tissue on the strainer using a 5 mL syringe plunger. Add another 500 µL NIB + 0.1% IGEPAL CA-630 and continue grinding tissue on the strainer. Move solution into a 1.5 mL microcentrifuge tube.
    NOTES:
    It is not necessary to push the whole tissue through the filter! Use your own discretion in deciding when to stop grinding the tissue through the filter, but make sure not to tear through the filter!
14. Transfer the isolated nuclei into 1.5 mL Lo-bind tubes and centrifuge at 500g, 4°C for 5 minutes.
15. Remove supernatants and wash nuclei with 1 mL NIB and centrifuge at 500g, 4°C for 5 minutes.
16. Remove supernatants and resuspend nuclei in ~900 µL NIB (final volume will be higher).
17. Take a few µL samples for counting using nuclear staining (e.g. DAPI). Adjust final concentration to ~5,000-10,000 nuclei/µL.
18. PERFORM THIS STEP IF YOU WISH TO STORE NUCLEI FOR LATER USE - OTHERWISE, SKIP TO THE SECOND PART OF THE NEXT STEP:

For long-term storage, resuspend nuclei in 100-500 µL NIB + 10% DMSO, and split them into 100 µL aliquots into 1.5 mL cryo tubes. Place into slow-freeze FreezeCell containers and stored at -80°C. Nuclei should be stored in liquid nitrogen to reduce degradation due to temperature fluctuations. (**STOP POINT**).

Nuclei Wash and filtering (~15 minutes for 4 samples)

19. PERFORM BELOW IF YOU ARE WORKING WITH PREVIOUSLY FROZEN, STORED NUCLEI:

    - Transfer nuclei from liquid nitrogen to 37°C water bath to thaw as quickly as possible. Place on ice immediately after being completely thawed.
    - Add 1000 µL NIB to each sample for washing and mix gently by vortexing.
    - Centrifuge nuclei at 500g, 4°C for 5 minutes, and remove supernatant by pipetting.

20. Add ~500 µL NIB and filter through a pluriStrainer Mini 40 µm filter into clean 1.5 mL LoBind tubes. Briefly centrifuge at 300g, 4°C for 15 seconds to let all the nuclei pass through.
21. Resuspend the nuclei in 100µL NIB.

Nuclei counting (~30 minutes for 4 samples)

22. Count the nuclei concentration for each sample.

    Optimally, use a buffer with DAPI and a fluorescent microscope to distinguish between actual nuclei and debris.

    Dissolve 10 mg DAPI in 2 ml of deionized water (dH2O) with a final concentration of 5 mg/ml. Split the DAPI solution into multiple tubes (100 ul per tube).

    Take out one tube (100 ul, 5 mg/ml DAPI), add 1.9 ml deionized water (dH2O). Split the diluted DAPI solution into multiple tubes (100 ul per tube, 0.25 mg/ml DAPI).
    Store the DAPI solution in the -20 °C freezer.

    Make the DAPI counting solution: in 500 µL of Nuclei Buffer, add 0.5 µL - 1µL of 0.25mg/mL DAPI solution.

    Take 1 µL of the sample and combine it with 9µL of the counting solution. Mix the solution and take 6 µL to dispense into a hemocytometer for counting the concentration of nuclei.

23. Adjust final concentration to 1000 nuclei/µL using NIB.

Tagmentation (~1 hour for 4 plates)

24. Dispense 5000 nuclei per well into 96-well plates by mixing the prepared nuclei with 2X TD buffer at 1:1 ratio.

    a. e.g. for each sample, mix 200 µL nuclei (1000 nuclei/µL) with 200 µL 2X TD buffer. Nuclei generally distributed into PCR strips and then distributed into wells - make sure not to pipet up and down in 96-plate wells to avoid introducing extra stress to the nuclei

25. Add 1 µL barcoded Tn5 enzyme in each well and cover with plastic foil.
26. Perform the tagementation at 55°C for 10 minutes with gentle shaking at 300 RPM.
27. Briefly spin down the plates (15 seconds) to collect all liquid from the sides.
28. Place on ice immediately to prevent over-tagmentation. Add 11 µL of 2X Stop Buffer. Mix gently by pipetting.

Pool/Centrifuge/Resuspend/Redistribute (30 minutes)

29. Pool nuclei and transfer them to 1.5 mL tubes.
30. Centrifuge the nuclei at 500g, 4°C for 5 minutes. Remove supernatant gently by pipetting. Leave a little excess to save as much nuclei as possible.
31. Wash nuclei with 1 mL NIB.
32. Centrifuge nuclei at 500g, 4°C for 5 minutes. Remove supernatant gently by pipetting.
33. Resuspend nuclei in 2150 µL NIB (1920 µL + ~10% excess for pipetting). Mix gently by vortexing.
    a. Notes:
       - Nuclei concentration can be counted at this step for sample loss calculations.
34. Load 5 µL resuspend nuclei (~5000 nuclei) into each well in 96-well plates.

a. Note: use 20 μL low retention tips, not wide-bore tips for higher pipetting accuracy.

Ligation (~2 hours for 4 plates)

35. To each well, add 2 μL DNA ligation primer/adaptor complex (3.3 μM).
36. To each well, add 3 μL T4 DNA ligase master mix in each well containing the following:
    a. 1 μL 10X T4 DNA Ligase Buffer
    b. 1 μL T4 DNA Ligase Enzyme
    c. 1 μL nuclease-free water
37. Cover plates with metal foil, and centrifuge briefly at 300g, 4°C for 15 seconds to collect all liquid from the sides.
38. Incubate plates for 30 minutes at room temperature with gentle shaking (300 rpm with Thermomixer, 50 rpm on Fisherbrand Nutating Mixer).
39. Place the plate on ice immediately and centrifuge briefly at 300g, 4°C for 15 seconds to collect all liquid from the sides.
40. Add 2 μL 18 mM EDTA to each well to stop the ligation reaction, pool all samples and transfer to 1.5 mL LoBind tubes.

Pool/Centrifuge/Resuspend/Redistribute/Quantify (30 minutes)

41. Centrifuge nuclei at 500g, 4°C for 5 minutes. Remove supernatant gently by pipetting. Leave a little excess to save as much nuclei as possible.
42. Wash nuclei with 1 mL NIB.
43. Centrifuge the tube at 500g, 4°C for 5 minutes. Remove supernatant gently by pipetting.
44. (Optional) Nuclei filtering.
    a. Resuspend nuclei in ~600 μL NIB and transfer them into a clean 1.5 mL LoBind tube.
    b. Filter the nuclei using a Pluristrainer Mini 5μm filter.
    c. Centrifuge nuclei briefly at 300g, 4°C for 15 seconds to allow all the nuclei to pass though.
    d. Wash the filter again 3 times with 100 μL NIB , followed by centrifugation at 300g, 4°C for 15 seconds to improve recovery rate. (~900 μL final sample)
45. Resuspend the nuclei in ~100 μL NIB, count nuclei and adjust the concentration to ~2000 nuclei/μL.
46. Distribute the nuclei into a 96 well plate with 10,000 nuclei per well.
    NOTES:
    - You can directly freeze and store cells at this point.
    - If you choose to freeze the plate, it is okay to place it directly in -80 °C freezer without flash-freezing
    - You can also store nuclei directly into PCR strips if you don't need to profile a whole plate of cells

Proteinase K treatment (overnight)

47. For each well, add 1 μL Proteinase K master mix:
    - 0.5 μL EB buffer
    - 0.25 μL 1% SDS
    - 0.25 μL Proteinase K (18.9 mg/ml)

48. Mix the samples by vortexing, followed by a brief centrifugation to collect all liquid from the sides, and vortex again to mix up the nuclei from the bottom.
49. Incubate samples at 65°C for 16 hours and keep at 4 °C until the following step.
50. Add 2 µL 10% Tween-20 to quench the SDS and mix well by pipetting.
    Note:
    - Adjust final volume to 8 µL using EB if sample evaporation occured in certain wells.
    - Samples can be stored at -20°C or -80°C until the PCR amplification step.

PCR Amplification (~1 hour)

51. Centrifuge the plates to collect all liquid from the sides.
    Notes:
    - We perform test qPCR runs on a few wells to determine appropriate PCR cycle number by adding dsGreen to the master mix.
    - Expected optimal cycle number is around mid-exponential phase, but higher cycle number is safer to use in order to ensure enough material for Novaseq sequencing.
    - Higher cycle number can increase yield, but it can also result in higher adapter dimer concentration.
52. To each well, add the following:
    - 1 µL 10 µM Universal P5 primer
    - 1 µL 10 µM Barcoded P7 primer
    - 10 µL NEBNext® High-Fidelity 2X PCR Master Mix.
53. Mix wells by pipetting. Centrifuge to collect all liquid from sides and remove bubbles.
54. Perform the following PCR reaction:
    a. 72°C for 5 minutes
    b. 98°C for 30 seconds
    c. 8 cycles of (98°C for 10 seconds, 66°C for 30 seconds, 72°C for 30 seconds)
    d. 72°C for 5 min
55. Store samples at -20°C until the sequencing library purification step (STOP POINT).

Sequencing Library Purification (1 hour)

56. Combine PCR products into a 1.5 mL tube.
57. Perform column purification using Zymo DNA Clean & Concentrator kit. Elute in 20 µL EB.
58. Run samples on 2% E-Gel™ EX Agarose Gels. Perform gel purification using Zymo Gel DNA Recovery Kit to remove primer dimers. Elute in 16 µL EB.
59. Quantify the library concentration and visualize the library via electrophoresis (performed using a Qubit and a 2% Agarose E-Gel). An example library is shown below:
60. Prepare Illumina sequencing library depending on the instrument recommendations:
    - NextSeq 1000: 2 nM DNA library, minimum 12 µL
    - NovaSeq 6000: 2.6 nM DNA library, minimum 310 µL
    Important: To increase library complexity, add minimum 20% PhiX before sequencing.
61. Sequence the library on the Novaseq Platform (Read1:100 bp, Read2: 100 bp, Index 1: 10 bp, Index 2: 10 bp).

NOTE: Perform a small-scale sequencing run before NovaSeq sequencing to prevent possible clustering and quality issues.

References

Hennig, Bianca P., Lars Velten, Ines Racke, Chelsea Szu Tu, Matthias Thoms, Vladimir Rybin, Hüseyin Besir, Kim Remans, and Lars M. Steinmetz. 2018. “Large-Scale Low-Cost NGS Library Preparation Using a Robust Tn5 Purification and Tagmentation Protocol.” *G3* 8 (1): 79–89.
