# Supplementary material for: A global view of aging and Alzheimer’s pathogenesis-associated cell population dynamics and molecular signatures in human and mouse brains
Source: Nat Genet. 2023 Nov 30;55(12):2104–16. doi: 10.1038/s41588-023-01572-y (PMC10703679; doi:10.1038/s41588-023-01572-y)
Supplement: Supplementary file 2 — Reporting Summary [file 41588_2023_1572_MOESM2_ESM.pdf]

nature portfolio

Corresponding author(s): Junyue Cao

Last updated by author(s): Sep 29, 2023

Reporting Summary

Nature Portfolio wishes to improve the reproducibility of the work that we publish. This form provides structure for consistency and transparency in reporting. For further information on Nature Portfolio policies, see our Editorial Policies and the Editorial Policy Checklist.

Statistics

For all statistical analyses, confirm that the following items are present in the figure legend, table legend, main text, or Methods section.

n/a	Confirmed	
☐	☒	The exact sample size (*n*) for each experimental group/condition, given as a discrete number and unit of measurement
☐	☒	A statement on whether measurements were taken from distinct samples or whether the same sample was measured repeatedly
☐	☒	The statistical test(s) used AND whether they are one- or two-sided<br>*Only common tests should be described solely by name; describe more complex techniques in the Methods section.*
☐	☒	A description of all covariates tested
☐	☒	A description of any assumptions or corrections, such as tests of normality and adjustment for multiple comparisons
☐	☒	A full description of the statistical parameters including central tendency (e.g. means) or other basic estimates (e.g. regression coefficient) AND variation (e.g. standard deviation) or associated estimates of uncertainty (e.g. confidence intervals)
☐	☒	For null hypothesis testing, the test statistic (e.g. *F*, *t*, *r*) with confidence intervals, effect sizes, degrees of freedom and *P* value noted<br>*Give P values as exact values whenever suitable.*
☒	☐	For Bayesian analysis, information on the choice of priors and Markov chain Monte Carlo settings
☒	☐	For hierarchical and complex designs, identification of the appropriate level for tests and full reporting of outcomes
☐	☒	Estimates of effect sizes (e.g. Cohen's *d*, Pearson's *r*), indicating how they were calculated

Our web collection on statistics for biologists contains articles on many of the points above.

Software and code

Policy information about availability of computer code

Data collection	No software was used except Illumina bcl2fastq/v2.19.0.316 for base calling.
Data analysis	Common,freely available sequencing data analysis software was used to analyze data,a s described in Methods:<br>python/v2.7.13, trim_galore/v0.4.1, STAR/v2.5.2b, bedtools/v2.30.0, R/v4.1, Seurat/v4.1, monocle/v2.22.0, Signac/v1.7.0, SnapATAC2/ v.1.99.99.3, scJoint/v1.0, umap/v0.5.3&v0.3.2, MACS2/v2.1.1, chromVar/v.1.16.0, chromVarmotifs/v0.2.0, motifmatchR/v.1.16.0, LDSC/v1.0.1.<br><br>Scripts for processing EasySci data was uploaded to github (https://github.com/JunyueCaoLab/EasySci) and have been referred in the manuscript.

For manuscripts utilizing custom algorithms or software that are central to the research but not yet described in published literature, software must be made available to editors and reviewers. We strongly encourage code deposition in a community repository (e.g. GitHub). See the Nature Portfolio guidelines for submitting code & software for further information.

Data

Policy information about availability of data

All manuscripts must include a data availability statement. This statement should provide the following information, where applicable:

- Accession codes, unique identifiers, or web links for publicly available datasets
- A description of any restrictions on data availability
- For clinical datasets or third party data, please ensure that the statement adheres to our policy

Processed and raw data can be downloaded from NCBI GEO (GSE212606) and have been referred in the data availability statement in the manuscript.

Research involving human participants, their data, or biological material

Policy information about studies with human participants or human data. See also policy information about sex, gender (identity/presentation), and sexual orientation and race, ethnicity and racism.

Reporting on sex and gender	We use the term sex throughout, and in our study this refers to genetic sex, determined by the composition of sex chromosomes. Balanced male and female samples were included in this study.
Reporting on race, ethnicity, or other socially relevant groupings	Socially relevent grouping were not taken into account to select the population of interest.
Population characteristics	Twelve individuals, including six controls and six alzheimer's disease patients ranging from 70 to 94 in age were used.
Recruitment	Samples were collected from the University of Kentucky AD Center Tissue Bank.
Ethics oversight	Each included participant who donated samples for this study signed a relevant consent form (including consent for unrestricted sharing of clinical, pathological, and genetic information for dementia research) that was approved by the UK Internal Review Board (UK IRB #44009).

Note that full information on the approval of the study protocol must also be provided in the manuscript.

Field-specific reporting

Please select the one below that is the best fit for your research. If you are not sure, read the appropriate sections before making your selection.

☒ Life sciences ☐ Behavioural & social sciences ☐ Ecological, evolutionary & environmental sciences

For a reference copy of the document with all sections, see nature.com/documents/nr-reporting-summary-flat.pdf

Life sciences study design

All studies must disclose on these points even when the disclosure is negative.

Sample size	In our previous single-cell genomic studies consisting of similar or lower numbers of replicates, we were able to detect significantly changed cell types with highly variable cell population sizes. For downstream analysis, we will in-silico sort and aggregate cells from the same cell type and individual, followed by differential expression analysis. We therefore anticipate that the sample size will provide sufficient power to detect significant change of cell types or DE genes in inter-condition analysis.
Data exclusions	No data were excluded from the study.
Replication	We confirm all attempts at replication were successful
Randomization	The experiments were carried out in a pooled manner.
Blinding	For collection brain tissues from different mice groups, blinding was not possible, since we need to record the genotype of each mice group and compare between conditions

Reporting for specific materials, systems and methods

We require information from authors about some types of materials, experimental systems and methods used in many studies. Here, indicate whether each material, system or method listed is relevant to your study. If you are not sure if a list item applies to your research, read the appropriate section before selecting a response.

Materials & experimental systems

n/a	Involved in the study
☒	☐ Antibodies
☐	☒ Eukaryotic cell lines
☒	☐ Palaeontology and archaeology
☐	☒ Animals and other organisms
☒	☐ Clinical data
☒	☐ Dual use research of concern
☒	☐ Plants

Methods

n/a	Involved in the study
☒	☐ ChIP-seq
☒	☐ Flow cytometry
☒	☐ MRI-based neuroimaging

Eukaryotic cell lines

Policy information about cell lines and Sex and Gender in Research

Cell line source(s)	HEK293T, NIH/3T3 cells were gift from Dr. Jay Shendure, University of Washington
Authentication	The cell lines were authenticated.
Mycoplasma contamination	Cell lines were not tested for Mycoplasma contamination.
Commonly misidentified lines (See ICLAC register)	No commonly misidentified cell lines were used.

Animals and other research organisms

Policy information about studies involving animals; ARRIVE guidelines recommended for reporting animal research, and Sex and Gender in Research

Laboratory animals	C57BL/6 wild-type mouse brains at three months (n=4), six months (n=4), and twenty-one months (n=4) were collected in this study together with 3 months old SxFAD (JAX stock #034840) (n=4) and APOE*4/Trem2*R47H (JAX stock #028709) (n=4) models. Two male and two female mice are included in each condition.
Wild animals	No wild animals were used.
Reporting on sex	Sex was determined by the composition of sex chromosomes. Both male and female mice were included in each condition. No sex-
Field-collected samples	No field-collected samples were used.
Ethics oversight	All animal procedures were in accordance with institutional, state, and government regulations and approved under the IACUC protocol 21049 and 20047.

Note that full information on the approval of the study protocol must also be provided in the manuscript.
